# A Randomized Controlled Trial Assessing Infectious Disease Risks from Bathing in Fresh Recreational Waters in Relation to the Concentration of *Escherichia coli*, Intestinal Enterococci, *Clostridium perfringens*, and Somatic Coliphages

**DOI:** 10.1289/ehp.8115

**Published:** 2005-09-29

**Authors:** Albrecht Wiedenmann, Petra Krüger, Klaus Dietz, Juan M. López-Pila, Regine Szewzyk, Konrad Botzenhart

**Affiliations:** 1 District Government Stuttgart, State Health Office, Stuttgart, Germany; 2 District Government Tübingen, Tübingen, Germany; 3 Department of Medical Biometry, University Hospital, Tübingen, Germany; 4 Federal Environmental Agency, Berlin, Germany; 5 Institute of General and Environmental Hygiene, University of Tübingen, Tübingen, Germany

**Keywords:** bathing, *Clostridium perfringens*, *Escherichia coli*, fecal indicators, fecal water pollution, fresh recreational water, gastroenteritis, health risks, intestinal enterococci, somatic coliphages

## Abstract

We performed epidemiologic studies at public freshwater bathing sites in Germany to provide a better scientific basis for the definition of recreational water quality standards. A total of 2,196 participants were recruited from the local population and randomized into bathers and non-bathers. Bathers were exposed for 10 min and had to immerse their head at least three times. Water samples for microbiological analysis were collected at 20-min intervals. Unbiased concentration–response effects with no-observed-adverse-effect levels (NOAELs) were demonstrated for three different definitions of gastroenteritis and four fecal indicator organisms. Relative risks for bathing in waters with levels above NOAELs compared with nonbathing ranged from 1.8 (95% CI, 1.2–2.6) to 4.6 (95% CI, 2.1–10.1), depending on the definition of gastroenteritis. The effect of swallowing water provided additional evidence for true dose–response relationships. Based on the NOAELs, the following guide values for water quality are suggested: 100 *Escherichia coli*, 25 intestinal enterococci, 10 somatic coliphages, or 10 *Clostridium perfringens* per 100 mL. Recreational water quality standards are intended to protect the health of those consumers who are not already immune or resistant to pathogens that may be associated with indicator organisms. In contrast to current World Health Organization recommendations, we concluded that standards should be based on rates of compliance with NOAELs rather than on attributable risks determined above NOAELs, because these risks depend mainly on the unpredictable susceptibility of the cohorts. Although in theory there is no threshold in real concentration–response relationships, we demonstrated that a NOAEL approach would be a more robust and practical solution to the complex problem of setting standards.

Recreational waters are monitored worldwide to protect the health of bathers from infectious diseases caused by waterborne pathogens that are associated with the pollution of natural recreational waters by human and animal feces, and the results of monitoring are the basis for corrective action where necessary. Water quality standards are typically based on measurements of the concentrations of fecal indicator organisms. In 1998 a review carried out on behalf of the World Health Organization (WHO) (Prüss 1998) assessed all available epidemiologic information on associations between the risk of infection for bathers and the concentration of fecal indicator organisms in fresh and marine recreational waters. Most studies reported an increase of health risk in swimmers with an increase in the indicator-bacteria count in recreational water. Relative risk (RR) values for swimming in polluted water versus clean water or versus staying on a beach without entering the water were often significant and usually ranged between 1 and 3. Both Prüss (1998) and the current guidelines of the WHO (2003) have identified the randomized controlled trial design as the one that yields the most reliable results when associations between the degree of fecal water pollution and the risk of infection are investigated. This conclusion has been based primarily on the fact that the randomized controlled trial design minimizes nondifferential misclassification bias resulting from the inaccurate assignment of microbial concentrations in the water to exposed participants. However, to this day, results from only one study of this kind are available for exposure to seawater. The results presented here are the first ever collected in a randomized controlled trial in fresh water. In addition, this randomized controlled trial is the first to study a cohort that includes children and teenagers, who usually make up a considerable percentage of the visitors at public bathing sites.

## Materials and Methods

### Study sites.

We performed the present study at five German freshwater bathing sites. All study sites had been registered to the European Commission’s atlas of European bathing beaches (European Commission 2005). They had complied with current European standards (Council of the European Communities 1976) for at least the three previous bathing seasons. Bathing sites in the European Union comply with the microbiological quality criteria when 95% of all sample results of a complete bathing season do not exceed the “imperative values” (legally binding) of 2,000 fecal coliforms and 10,000 total coliforms per 100 mL. In addition, it is recommended that 80% of the sample results comply with a “guide value” of 100 fecal coliforms and that 90% of the samples comply with a guide value of 100 fecal streptococci per 100 mL.

Study locations were situated in the north, northeast, southwest, and southeast of Germany: four sites on lakes and one site on a river. Sources of fecal contamination included treated and untreated municipal sewage, agricultural runoff, and contamination from water fowl [details in Supplemental material Annex 1 (http://ehp.niehs.nih.gov/docs/2005/8115/supplemental.pdf)].

### Ethical clearance.

The trial design was approved by the ethics commission of the medical faculty of the Eberhard Karls University Tübingen.

### Recruitment of the study cohort.

A total of 2,196 participants were recruited from the local population. In the 3 weeks before each trial, the study design and the purpose of the study were promoted with the help of the local media, and information flyers were distributed at information desks and on the beaches. People could register for participation either directly at the information desks or by calling a telephone number and making an appointment for an initial interview and medical examination. For the pilot trial at study location 1, only volunteers > 18 years of age were accepted for participation. After additional ethical clearance, the lower age limit was set to 4 years for the remaining trials at study locations 2–5. All adult participants and teenagers between 14 and 18 years of age gave their written informed consent before participating. Teenagers between 14 and 18 years of age were accepted with the written, signed consent of their parents or legal guardians. Children < 14 years of age were accepted with the written, signed consent of both parents or legal guardians and had to be accompanied by at least one parent or a legal guardian. Children < 4 years of age were not accepted because they were considered to be a potential source of fecal accidents during the trials.

### Enrollment.

Two to three days before exposure, the participants were interviewed in person, and all participants underwent a short medical examination. The medical checkups were performed by professional physicians and included monitoring the questionnaire data with respect to the details on the participants’ health conditions; a brief discussion on specific health problems such as chronic diseases, infections, or injuries in the participant’s medical history if required; an inspection of the eyes, ears, and throat; and an electronic temperature measurement in the ear. Volunteers were included if they appeared to be physically and mentally fit for participation in the study trials. Reasons for exclusion were serious acute infectious diseases or fever (> 38.0°C), acute nausea or diarrhea, wounds that had not healed, or health conditions where bathing might have presented a serious or life-threatening risk.

### Allocation.

On the exposure day, participants were randomized into equal-sized groups of bathers and nonbathers. Randomization took place after registration at a registration desk on the beach using a block randomization procedure with blocks of 10. Parents of children < 14 years of age were asked whether their children would accept a randomization result independent of the result of their parents. If the answer was yes, the children were independently randomized. If children preferred to be accompanied by one of their parents, they had to decide by which one. Parents were also given the option of making this decision for their children. Subsequently, the parents received their randomization result.

### Trial design.

Each participant received a standardized lunch package and a bottle of mineral water; 20 of the lunch packages per trial were microbiologically analyzed and monitored for food pathogens in a state food safety laboratory. All food items complied with their specific quality standards. The purpose of serving this controlled lunch package was to exclude the theoretical possibility of a food-borne outbreak that might have occurred if the participants had bought their lunch at local fast-food stores on the beach.

The bathers were assigned to one of four roped-off bathing areas of approximately 10 m width and 20 m length that were distributed across the beach, with each divided into a non-swimmers’ zone with shallow water and a swimmers’ zone. The nonbathers were directed to a roped-off area of the beach with lawn or sand grounds where they could not come into contact with the water.

When the participants arrived at their destination—either the nonbathers’ area or one of the four bathing areas—they were interviewed again by one of the project helpers. In this second interview, the questions focused on symptoms having occurred after the first interview and on nutritional details of the preceding 2 or 3 days. Participants who reported any new symptoms were sent to one of the physicians on site, who decided whether these symptoms were a reason for exclusion. After the interview, the participants were free to have their lunch, and nonbathers were free to stay in the non-bathers’ area for normal beach activities such as sunbathing or playing, depending on the weather conditions.

Participants who had been randomized for bathing entered the water under the individual supervision of their interviewers. Bathing duration was limited to exactly 10 min, and participants were asked to stay inside the roped-off swimming zones and to stay or swim around balloons that had been placed like buoys in the center of the zones. They were also instructed to completely immerse their heads at least three times during the 10 min. The supervisors individually recorded the start and ending times of the bathing period and the number of head immersions performed in each of the 10-min-periods. After leaving the water, the participants were asked whether they had accidentally swallowed water.

### Microbiological analyses.

For the entire time the participants were being exposed, water samples were collected every 20 min from the centers of the swimmers’ and nonswimmers’ zones in all four areas. These samples were analyzed in a nearby mobile laboratory for six microbiological parameters: *Escherichia coli* [International Standardization Organization (ISO) 1998b], intestinal enterococci (ISO 1998a), *Clostridium perfringens* (Council of the European Union 1998), somatic coliphages (ISO 2000), aeromonads (Schulze 1996), and pyocyanine-positive *Pseudomonas aeruginosa* (Deutches Institut für Normung, European Normalization 2002). The values of the method-specific lower detection limits were assigned to all samples with analytical results below the detection limit, and the results were censored. Upper detection limits were not reached in any of the samples. Method details are given in Supplemental material Annex 2 (http://ehp.niehs.nih.gov/docs/2005/8115/supplemental.pdf).

### Microbiological quality control.

Quality control procedures included positive and negative media controls for all target organisms and temperature control of all incubators with continuously operating digital temperature displays and additional electronic devices recording minimum and maximum temperatures throughout the incubation period. As a quality control procedure for *E. coli* and intestinal enterococci, external quantitative reference materials were applied that had been evaluated in earlier international round-robin trials (“reference lenticules K,” donated by Institut Pasteur de Lille, Lille, France; European Community contract SMT4-CT95-1603/DG12-RSMT; Contreras-Coll et al. 2002). PhiX-174 coliphages were used as positive controls in the somatic coliphage assay.

### Follow-up.

One week after exposure, all the participants were interviewed again in person and underwent a medical inspection of the throat, eyes, and ears. Interviewers and doctors were unaware of the exposure status of the participants. Three weeks after exposure, the participants received a last questionnaire by mail. After receipt of this fourth questionnaire, each participant received a compensation of 25 Euros to cover personal expenses.

### Data entry, verification, and analysis.

All questionnaire data were entered into an electronic database created with Epi Info (version 6.2; Centers for Disease Control and Prevention, Atlanta, GA, USA) and verified by an independent second entry. Statistical analyses were performed using the JMP (version 5.0; SAS Institute Inc., Cary, NC, USA), STATCALC in Epi Info, and MS Excel 97 (Microsoft Corporation, Redmond, WA, USA).

### Calculation of individual exposure concentrations.

For each minute of trial duration (between 220 and 240 min, depending on the number of participants per site), microbial concentrations in the water were calculated by arithmetic interpolation between the results obtained by analyzing the water samples. This was done for all bathing areas and in both the swimmers’ and the nonswimmers’ zones. The microbial concentrations of each of the 10 min of water contact were individually assigned to each of the bathers in their individual exposure area. The values from the nonswimmers’ or the swimmers’ zones were used, depending on where the participant was staying. Finally, individual exposure concentrations for all microbiological parameters were assigned to each bather by calculation of the arithmetic mean concentration of the 10 individual exposure minutes. Participants with unacceptable exposure data were excluded from further analysis. Exposure data were considered to be unacceptable if the participants did not comply with their randomization status, if they entered the water too early or too late, or if the total exposure time was less or more than 10 min.

### Exposure definitions.

We defined exposure in two different ways. The first definition was “10 min bathing with at least three head immersions,” which is equivalent to the instructions that the participants had received from the study organizers. For this definition the arithmetic mean of the 10 concentrations that had been calculated and assigned as described above was used as the mean exposure concentration of every individual bather.

We used the second definition of exposure to assess the influence of the number of head immersions. It takes into account the fact that the participants followed the instructions to immerse their heads at least three times to a varying extent. Because each immersion of the head could be looked on as an equivalent for the uptake of a certain small amount of water via the eyes and the lacrimal duct, the nose and throat, and the mouth, the minute-specific concentrations of all minutes during which the head was immersed were multiplied with the number of head immersions during each of these minutes and added. The result can be considered to be the theoretical equivalent of a single head immersion at that concentration. This second theoretical definition of exposure was therefore called “single head immersion.”

### Calculation of incidence rates, RRs, and attributable risks.

We calculated incidence rates as the number of cases with onset of disease in the week or the 3 weeks after exposure divided by the number of individuals observed during these periods in a defined exposure category [nonbathers, bathers, bathers exposed below or above no-observed-adverse-effect levels (NOAELs), bathers exposed in quartile or quintile categories of microbial concentrations], and used percentage as the unit of measurement. We calculated RRs as the incidence rates among participants in a defined exposure category (bathers or bathers exposed above NOAELs) divided by the incidence rates among unexposed participants (nonbathers). We calculated attributable risks (aRs) as the difference between the incidence rates (percent) observed in two different exposure categories (e.g., incidence rates of bathers above NOAELs minus the incidence rates of non-bathers). Initially, we compared the crude incidence rates of disease among bathers and nonbathers, the crude RRs, and the crude aRs after the 1-week evaluation interval with those after the 3-week interval. After it became obvious from this comparison that there was no additional scientific benefit from using the 3-week interval, all subsequent analyses concentrated on the results obtained within a period of 1 week.

### Determination of NOAELs.

To determine potential NOAELs, we sorted participants by their individual exposure concentration in ascending order. We then performed a Pearson’s chi-square test for every actually occurring concentration to compare the incidence rates among bathers exposed below and above this concentration, and the resulting *p*-value was recorded. The most probable estimate for a potential NOAEL was considered to be the concentration that revealed the lowest *p*-value, provided that the following conditions were met: *a*) the incidence rate among bathers above this concentration was significantly higher than the incidence rate among bathers below this concentration (*p* < 0.05); *b*) the incidence rate of bathers below this concentration was not significantly lower than the incidence rate of nonbathers (exclusion of a coincidental imbalance in the distribution of cases); and *c*) no expected cell value was less than five (exclusion of unreliable test results). The reliability of this procedure was tested with sets of simulated data with defined dose–response relationships.

### Control for bias.

We tested potential NOAELs against a variety of possible confounding variables in the total cohort, including age, sex, study location, weather conditions, accidental swallowing of water during the trial, previous or additional water contact or water-related activities, previous diseases, diseases in household members, other household members participating in the study, consumption of prescription drugs, various nutritional factors, consumption of alcohol and tobacco, travel history, socioeconomic status, leisure activities, risk perception, membership in environmental organizations, and background information on recreational water monitoring. Initially, all variables were univariately screened for possible effects in the total final cohort of bathers and nonbathers (Pearson’s chi-square tests). Variables showing significant univariate effects (*p* < 0.05) were considered to be potential confounders of the NOAELs and were further analyzed using a multiple logistic regression procedure (effect likelihood ratio test) in which the disease was modeled as the response variable, and the potential NOAEL and the potential confounder as model effects. If NOAELs remained significant effects in these tests (*p* < 0.05), the NOAELs were considered to be unbiased, and the potential confounder was considered to be an independent predictor of disease. In addition, all models were analyzed for possible interaction effects between NOAELs and potential confounding variables by crossing both effects in separate effect likelihood ratio tests. Significant interaction effects (*p* < 0.05) were recorded but NOAELs were not rejected. We testesd all potential NOAELs and the possible confounding variables one by one to exclude effects caused by a possible collinearity of the indicator organisms. Stratified analysis as a possible alternative method to test for confounding was considered inappropriate because of the limited number of cases and the loss of test power that would have been associated with stratification.

In addition, we compared potential NOAELs with the results of classifying bathers into quartile and quintile categories of exposure concentrations. Potential NOAELs were considered acceptable and unbiased estimates for a true NOAEL if they remained significant model effects (*p* < 0.05) in all effect likelihood ratio tests and if none of the incidence rates in quartile or quintile categories with upper range limits below the potential NOAEL was significantly higher than the incidence rate of the nonbathers.

We evaluated the influence of the accidental swallowing of water and the plausibility of the NOAELs by categorical analysis of the incidence rates of disease in bathers exposed below and above NOAELs.

### Disease definitions.

We analyzed data for the following diseases as outcome variables: acute febrile respiratory infections, common cold, ear inflammation, eye inflammation, skin infections or cutireactions, urinary tract infections, and finally three more or less stringent definitions of gastroenteritis: *a*) definition “GE_UK” according to Kay et al. (1994), *b*) definition “GE_UK-wf” according to the present study, and *c*) definition “GE_NL-2” according to van Asperen et al. (1998).

We defined a case of gastroenteritis by Boolean combination of symptom variables from the questionnaire as follows (Boolean operators in capital letters): GE_UK, (diarrhea AND three or more bowel movements per day) OR vomiting OR (nausea AND fever) OR (indigestion AND fever) [Note: there were no occurrences of (nausea AND fever) or (indigestion AND fever)]; GE_UK-wf, that is, GE_UK without consideration of stool frequency: diarrhea OR vomiting OR (nausea AND fever) OR (indigestion AND fever) [Note: there were no occurrences of (nausea AND fever) or (indigestion AND fever)]; GE_NL-2, diarrhea OR nausea OR vomiting OR stomach pains.

## Results

### Participant flow.

**Figure 1:**
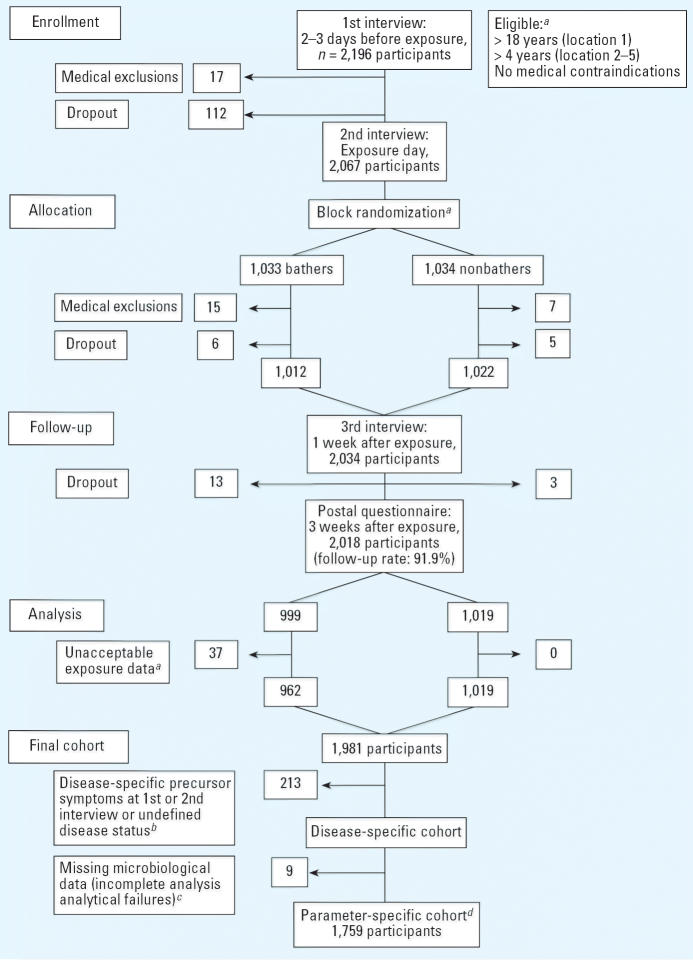
Participant flow through each stage of the trial in CONSORT format (Moher et al. 2001) for the analysis of gastroenteritis (definition GE_UK-wf) and the concentration of *E. coli* and intestinal enterococci. ***^a^***Details are explained in “Materials and Methods.” ***^b^***Number depends on the disease definition. ***^c^***Number depends on the microbiological parameter. ***^d^***Number depends on the disease definition and the microbiological parameter.

The participant flow through each stage of the trial is displayed in Figure 1 in CONSORT format (Moher et al. 2001). The follow-up rate was 91.9%; that is, 2,018 of initially 2,196 participants returned the last questionnaire.

### Cohort characteristics.

The age distribution among bathers and nonbathers in the final cohort of 1,981 participants was almost identical. Minimum, 25th percentile, median, 75th percentile, and maximum in the group of bathers were 4, 14, 23, 39, and 79 years versus 4, 15, 25, 39, and 89 years in the group of nonbathers. The ratios of male to female participants in the group of bathers were 50.8:49.2% and 46.5:53.5% in the group of nonbathers; 1.7% of the participants were preschool children, 33.4% were schoolchildren, 11.9% were students, 35.9% were employed, 7.6% were homemakers or retired, 5.2% were unemployed, and 4.3% did not disclose their employment status; 7.1% were members of an environmental organization.

### Exposure intensity.

Only 9% of the participants immersed their heads less often than required, whereas 53% voluntarily did it more often (median, 4; mean, 5.5; range, 0–87). High numbers of head immersions were recorded for some well-trained swimmers who immersed their head with almost every swimming movement.

### Disease-specific cohorts.

Disease-specific cohorts were formed by exclusion of participants who reported disease-specific precursor symptoms at the first or second interview or whose disease status was undefined. Disease-specific precursor symptoms used to evaluate the risk of acquiring gastroenteritis were considered to be any of the following symptoms: fever, loss of appetite, nausea, vomiting, stomach pains or cramps, indigestion, loose bowel movements, or diarrhea. For the evaluation of other disease risks, specific precursor symptoms were chosen accordingly. The disease status was considered to be undefined if any of the symptom variables that were required to define a case was missing or answered by “not sure.” For the analysis of gastroenteritis, 188 participants were excluded because of typical precursor symptoms. In addition, the following numbers of participants were excluded because of their undefined disease status, depending on the definition of gastroenteritis: 36 (GE_UK), 25 (GE_UK-wf), or 26 (GE_NL-2).

### Parameter-specific cohorts.

From the disease-specific cohorts, parameter-specific cohorts were formed by exclusion of data sets with missing microbiological data for each of the microbiological parameters. For the analysis of gastroenteritis, the following number of data sets had to be excluded depending on the kind of fecal indicator organism: none (*C. perfringens*), 9 (*E. coli* and intestinal enterococci), or 12 (somatic coliphages). Thus, the parameter-specific cohort sizes for the analysis of gastroenteritis ranged between 1,745 and 1,768.

### Microbial concentrations in the water.

The total number of available sample results per parameter, the median concentrations, and the correlation coefficients between the microbiological parameters are listed in Supplemental material Annexes 2 and 3 (http://ehp.niehs.nih.gov/docs/2005/8115/supplemental.pdf). The concentration ranges for the fecal indicators were as follows: *E. coli*, 4.7–5,344/100 mL; intestinal enterococci, 3.0–1,504/100 mL; *C. perfringens*, 9–260/100 mL; somatic coliphages, 10–3,780/100 mL. The lower range limits are equivalent to the lower detection limits. Upper detection limits were not reached in any of the samples.

### Microbiological quality control.

The results obtained from the quantitative quality control tests for *E. coli* and intestinal enterococci using “reference lenticules K” were well within the range of results that could be calculated in a round-robin trial performed by nine European expert labs as described above. The means and the standard deviations of the log_10_-transformed results obtained during the study trials were 2.76 ± 0.08 *E. coli*/100 mL and 2.47 ± 0.09 intestinal enterococci/100 mL (*n* = 38) compared with 2.77 ± 0.18 *E. coli*/100 mL and 2.49 ± 0.30 intestinal enterococci/100 mL (*n* = 43) obtained in the round-robin trial.

### Compliance of the study sites with current European Union standards.

In 95.2% of the samples (401 of 421) collected during the 5 trial days, *E. coli* concentrations were below the imperative value of 2,000/100 mL. Thus, in the time periods during which the trials were performed, the total water quality of all five study locations would have just passed the current European standard (95% of sample results < 2,000/100 mL).

### Crude incidence rates and RRs of bathers versus nonbathers.

Significant or borderline significant differences between the crude incidence rates of bathers versus nonbathers could be observed only for gastroenteritis 1 week after exposure and for skin ailments. The incidence rates of gastroenteritis in the nonbathers group (“baseline risks”) corresponded well with the three different definitions of gastroenteritis, with the most stringent definition (GE_UK) revealing the lowest rate of 1.4% [95% confidence interval (CI), 0.8–2.4] and the less stringent definitions (GE_UK-wf and GE_NL-2) revealing higher rates of 2.8% (95% CI, 1.9–4.1) and 5.2% (95% CI, 3.9–6.9). The same effect could be observed in the group of bathers. Crude aRs of gastroenteritis slightly decreased after 3 weeks, indicating that virtually all episodes of bathing-associated gastroenteritis occurred within an incubation period of 1 week and that symptoms were less likely to be remembered for a time period of 3 weeks than for a period of 1 week. Further data evaluation therefore focused on the results obtained after 1 week. Detailed figures are displayed in Supplemental material Annex 4 (http://ehp.niehs.nih.gov/docs/2005/8115/supplemental.pdf).

### NOAELs, incidence rates, RRs, and aRs.

NOAEL estimates that were unbiased by any of the potential confounding variables could be determined for all three definitions of gastroenteritis and all four fecal indicator organisms, whereas the concentrations of aeromonads and *Pseudomonas aeruginosa* were not associated with gastroenteritis in any case. Detailed descriptions of the 160 tested variables are given in Supplemental material Annex 5(http://ehp.niehs.nih.gov/docs/2005/8115/supplemental.pdf). Details of the results of the effect likelihood ratio tests are recorded in Supplemental material, Annexes 6 and 7 (http://ehp.niehs.nih.gov/docs/2005/8115/supplemental.pdf). NOAELs for nonenteric diseases were either nonexistent or nondetectable with the available cohort size or within the range of microbial concentrations encountered during the present study; in the case of the common cold, they were confounded by other risk factors. A possible partial association between the concentration of aeromonads and skin ailments (cutireactions) was detectable, but it was overlaid by an unknown independent water-related effect; that is, there must have been other causes for cutireactions among bathers that were not associated with the microorganisms monitored in the present study. Because all NOAELs were above the lower detection limits of the microbiological methods, there was no need for application of special techniques to account for censored data below the detection limits.

The incidence rates, RRs, and aRs for gastroenteritis below and above the potential NOAELs, the incidence rates and aRs depending on the accidental swallowing of water, and the corresponding raw numbers are listed in detail in Supplemental material Annex 8 (http://ehp.niehs.nih.gov/docs/2005/8115/supplemental.pdf). Table 1 summarizes NOAELs, RRs, and aRs for all combinations of exposure definitions, definitions of gastroenteritis, and fecal indicator organisms. For exposure definition 1 (“10 min bathing with at least three head immersions”), NOAELs of the most widely used fecal indicators *E. coli* and intestinal enterococci, for example, ranged between 78 and 180 *E. coli*/100 mL and between 21 and 24 intestinal enterococci/100 mL. RRs for these two indicators ranged from 1.9 (95% CI, 1.3–2.8) to 3.5 (1.8–7.0), and aRs for bathing above NOAELs ranged from 3.1 to 5.0% depending on the definition of gastroenteritis.

Swallowing water compared with not swallowing water resulted in significantly higher aRs above NOAELs than below NOAELs [arithmetic mean aRs: 3.6% vs. 1.3%; *p* < 0.0001, analysis of variance (ANOVA)]. In addition, swallowing water below NOAELs never resulted in any significant effect compared with nonbathing, whereas swallowing water above NOAELs always revealed significant effects (all *p*-values < 0.003), thus providing evidence for true dose–response relationships.

**Figure 2:**
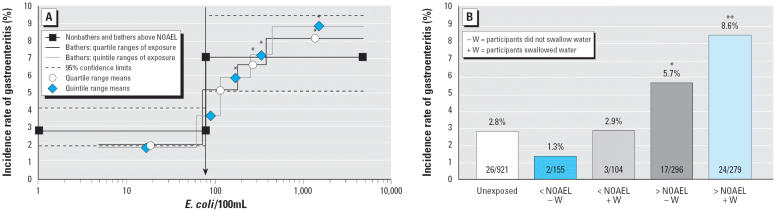
Incidence rates of gastroenteritis (definition GE_UK-wf) within 1 week after exposure to fresh recreational water in quartile and quintile categories of *E. coli* concentrations (*A*) and incidence rates below and above the presumed NOAEL of 78 *E. coli* /100 ml (*B*), depending on the accidental swallowing of water. *p < 0.05 in a chi-square test or Fisher’s exact test comparing the incidence rate of gastroenteritis in one of the specified exposure categories with the incidence rate of gastroenteritis in the group of unexposed participants (nonbathers). **p < 0.001.

The results from classifying bathers into quartiles and quintiles of the exposure concentrations generally corresponded well with the determined NOAELs. The incidence rates of gastroenteritis in the first quartile and in the first quintile were below the upper limit of the 95% CI of the incidence rate of nonbathers for all 24 combinations of exposure definition, definition of gastroenteritis, and fecal indicator organism. Pearson’s chi-square tests did not reveal a significant difference between the incidence rates of nonbathers and the incidence rates in the first quartile or quintile in any of the 24 combinations. On the other hand, all incidence rates within the fourth quartile and, with only one exception, all incidence rates within the fifth quintile were within the 95% confidence intervals of the incidence rates of bathers above NOAELs. For most quartiles and quintiles with concentration ranges above NOAELs, Pearson’s chi-square tests or Fisher’s exact tests revealed significant differences between the incidence rates within the quantiles and the incidence rates among non-bathers. Only the presumptive NOAEL of 150 somatic coliphages/100 mL for GE_UK and exposure definition 1 was obviously too high and seems to mark the level where the concentration–response curve reaches its maximum rather than the true NOAEL. A complete list with incidence rates of gastroenteritis in quartile and quintile categories of fecal indicator concentrations for the three different definitions of gastroenteritis and the two exposure definitions is given in Supplemental material Annexes 9 and 10 (http://ehp.niehs.nih.gov/docs/2005/8115/supplemental.pdf). An example with incidence rates in quartile and quintile categories of microbial exposure concentrations for GE_UK-wf is given in Table 2. The charts in Figure 2 demonstrate a comparison of the incidence rates of gastroenteritis in quartile and quintile categories of *E. coli* concentrations and the incidence rates below and above the NOAEL depending on the accidental swallowing of water for GE_UK-wf.

### Influence factors on NOAELs, RRs, and aRs.

The NOAELs depended mainly on the definition of exposure, with significantly higher values for exposure definition “single head immersion” (*p* = 0.009, ANOVA for log_10_-transformed data). They also depended on the microbiological parameter (*p* = 0.006). They did not, however, depend on the definition of gastroenteritis (*p* = 0.32).

Like the incidence rates of nonbathers and the incidence rates of bathers exposed below NOAELs, the incidence rates of bathers above NOAELs mainly depended on the definition of gastroenteritis (*p* < 0.001). There was no significant difference between the two exposure definitions (*p* = 0.90) and between the four fecal indicator parameters (*p* = 0.88).

The mean risk attributable to swallowing water above NOAELs was significantly higher than that attributable to swallowing water below NOAELs [3.6 (range, 1.6–8.6) vs. 1.3 (range, −0.4 to 3.8); *p* < 0.001, ANOVA].

### Severity of bathing-associated gastroenteritis.

The RRs were higher for the more stringent definitions of gastroenteritis. Mean RRs of bathers above NOAELs versus nonbathers were 3.7 (range, 3.12–4.61) for GE_UK, 2.6 (range, 2.43–2.78) for GE_UK-wf, and 1.9 (range, 1.71–2.33) for GE_NL-2. Mean aRs above NOAELs were 3.8% (range, 3.1–5.2), 4.4% (range, 4.2–5.7), and 4.7% (range, 4.0–8.6), respectively. This demonstrates that most gastroenteritis cases attributable to bathing were cases meeting the most stringent definition of gastroenteritis; that is, they were not simply mild forms of gastroenteritis. Two of the 26 nonbathers (7.7%; 95% CI, 0.9–25.1) who developed gastroenteritis (GE_UK-wf) within 1 week after the trial day consulted a doctor, versus 3 of the 40 bathers (7.6%; 95% CI, 1.6–20.4) who were exposed above NOAEL. Thus, there was no indication that waterborne gastroenteritis was less severe than gastroenteritis acquired through other sources of infection.

## Discussion and Conclusions

The rationale behind the use of indicator organisms since their introduction into water hygiene more than 100 years ago was simply the empirical observation that below certain concentrations—below certain extents of fecal pollution—the disease risk from certain fecal–orally transmittable pathogens was negligible. It has never been assumed that certain concentrations of indicator organisms above these levels are inevitably associated with certain predictable risks. Fecal indicator organisms such as *E. coli* and intestinal enterococci are part of the normal bacterial flora of the guts of all warm-blooded animals, including human beings. With only rare exceptions (e.g., enterohemorrhagic *E. coli*), they do not cause disease. In food matrices where they can multiply, microorganisms of fecal origin may be of only little value as indicators of health risks. According to federal law in Switzerland, cheese may contain up to 10,000 *E. coli* per gram (Eidgenössisches Volkswirtschaftsdepartement 1999). In recreational waters, fecal indicator organisms can be associated with varying amounts and changing kinds of pathogens, depending on the spread of infection at the source of the fecal pollution, and the susceptibility of exposed cohorts is highly variable and hardly predictable. Therefore, the assumption that the risk of acquiring gastrointestinal illness from exposure to certain concentrations of fecal indicator organisms is predictable, as implied by recent publications (Havelaar et al. 2001; Kay et al. 1994, 2004), may be true only for a defined population under the condition of a constant degree of endemicity of always the same kinds of pathogens. It would not, however, be a sufficient basis for the derivation of generally applicable microbiological standards for fecal indicator organisms in water. According to Prüss (1998), maximum detectable aRs of gastroenteritis from exposure to recreational water ranged between 0.4 and 27.7% in studies involving freshwater exposure, and from 0.5 to 19.5% in studies involving seawater exposure. A comparison of the results from randomized exposure to fresh water (present results) with the results from randomized exposure to seawater (Kay et al. 1994) confirms that different aRs (4.5% vs. 19.5%) can occur in two different cohorts (Germany vs. United Kingdom) above almost identical NOAELs [24 intestinal enterococci/100 mL in the present study vs. 32 fecal streptococci/100 mL reported by Kay et al. (1994)] even if the type and intensity of exposure are nearly the same (10 min bathing, three or more head immersions). If the pathogen: indicator ratios in the German and U.K. studies had been completely different, this would most likely have caused larger differences between the NOAELs. The most probable explanation for the differences in the baseline risks (nonbathers) and the aRs (bathers above NOAELs) observed in the German and the U.K. trials are differences in the cohort susceptibilities (Dizer et al. 2005).

If the determination of NOAELs is based on observable differences between groups—nonbathers, bathers below NOAEL, and bathers above NOAEL—then NOAEL is also a function of statistical significance, which is a function of sample size and of the susceptibility status of the population. A possible explanation for the slightly lower NOAELs in the German trial may therefore be the relatively larger cohort size (1,748 vs. 1,216) and the relatively lower background rate of disease in the group of nonbathers (4.8% vs. 9.7%; GE_UK, each within 3 weeks after exposure) and in the group of bathers below NOAEL (4.4% vs. 10.9% among bathers exposed at concentrations of up to 20 intestinal enterococci or fecal streptococci per 100 mL). On the other hand, the lower susceptibility of the German cohort may have partially compensated this effect.

If recreational water standards are based on predefined maximum “acceptable” disease risks for bathers (or estimates of the “disease burden” from bathing) as identified in a certain epidemiologic study, the resulting values for “acceptable” concentrations of fecal indicator organisms mainly depend on three variables: *a*) the NOAEL detected in that study, *b*) the susceptibility of the cohort in that study, and *c*) the definition of the disease. NOAELs mainly depend on the pathogen:indicator ratio (the higher the ratio the lower the NOAEL) and on the intensity of exposure (the more intense the exposure the lower the NOAEL). The measurable susceptibility of a cohort depends on the degree of immunity—the percentage of individuals who can be infected—and on the percentage of infected individuals who develop symptoms, because only symptomatic disease will be reported. The definition of gastroenteritis (more or less stringent) does not affect the NOAEL; however, it significantly affects the baseline level of gastroenteritis in nonbathers and bathers below the NOAEL, the RR, and the aR.

It has been proposed that imperative (legally binding) values for fecal indicator concentrations in recreational water standards be set at a level that limits the aR of acquiring gastroenteritis to 5% and that guide (recommended) values should be set at a level that limits the aR to 3% (Havelaar et al. 2001; Kay et al. 2004). This concept has been applied in the current revision of the European bathing water directive [Commission of the European Communities (CEC) 2002] using both the NOAEL and the aR depending on the fecal indicator concentration above the NOAEL as determined in the Kay et al. (1994) studies at British seawater bathing sites. These studies revealed an overall aR of approximately 9.5% above a concentration of 26 fecal streptococci/100 mL. This is about three times more than we have observed in the German trials above a concentration of 24 intestinal enterocci/100 mL using the same definition of gastroenteritis (GE_UK).

**Figure 3:**
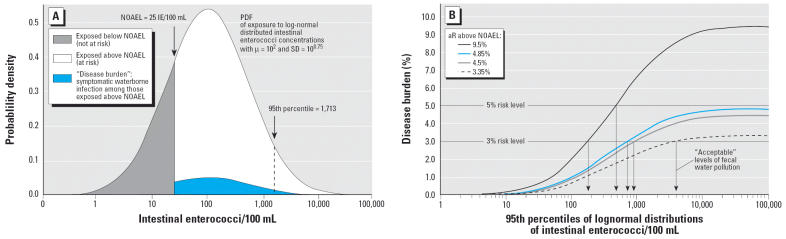
Calculation of the disease burden from exposure to log-normal distributed intestinal enterococci concentrations (*A*) and the influence of cohort susceptibility and disease definition on the acceptable level of fecal water pollution if standards are based on predefined attributable risks (in this case, 3 and 5%) above an assumed identical NOAEL of 25 intestinal enterococci/100 mL (*B*). Details on the calculation of the PDF, the disease burden, and the aRs are explained in “Discussion.”

The reason why the concept of basing water quality standards mainly on aRs is less robust than basing them solely on NOAELs is demonstrated in Figure 3, which shows the effect of different cohort susceptibilities (British vs. German cohort) and different definitions of gastroenteritis (GE_UK, GE_UK-wf, and GE_NL-2) on the “acceptable” level of fecal water pollution if standards are based on predefined “acceptable” aRs (5% or 3%) above an identical NOAEL. The probability density function (PDF) in Figure 3 was calculated using a geometric mean value (μ) of 100 intestinal enterococci/100 mL, which is the current European guide value for 90% of the sample results (Council of the European Communities 1976) and also the proposed value for the 95th percentile in the proposal for a revised guideline (CEC 2002). The standard deviation was chosen according to similar calculations that have been used in recommendations of the WHO (Kay et al. 2004). The “disease burden” associated with exposure to recreational water with fecal indicator concentrations that vary according to a PDF with a given μ and an SD of 10^0.75^ (which can also be characterized by its corresponding 95th percentile) was calculated according to the following equation: disease burden = [1 − *p*(cumulative PDF)] × (mean aR above NOAEL). This formula represents a simplification of the WHO concept, which uses a hypothetical square root function to describe a successive increase in aR above the NOAEL (32 fecal streptococci/100 mL) and a constant risk above the upper range limit of experimental data (158 fecal streptococci/100 mL) (Kay et al. 1994, 2004). The aRs (bathers’ excess risks) that were used in the sample calculations in Figure 3 were chosen on the basis of results from studies with a randomized controlled study design and an exposure intensity characterized by 10 min of bathing with three or more head immersions: 9.5% is the aR that was observed in the trials performed at British sea-water bathing sites (Kay et al. 1994) in the group of bathers exposed at fecal streptococcus concentrations of 26/100 mL or higher (British cohort); 3.35, 4.5, and 4.85% are the arithmetic means of the aRs associated with exposure above the presumptive NOAELs for *E. coli* and intestinal enterococci that could be observed at German freshwater bathing sites (German cohort; present results) for three more or less stringent definitions of gastroenteritis.

These calculations demonstrate that, despite the clear effects that we have found, it would be impossible to set imperative values for German freshwater bathing sites based on the results of the present study, and that guide values for compliance at 95th percentile levels of monitoring results for intestinal enterococci would range from approximately 200 to approximately 4,000/100 mL depending on nothing else but different cohort susceptibilities in the German and the U.K. trials and on different definitions of gastroenteritis. If the concept of basing regulatory standards on a maximal acceptable aR of 5% and 3% were applied to studies other than those performed by Kay et al. (1994), no imperative or guide values would be necessary at all—for example, for Egyptian beaches (Cabelli 1983) or Hong Kong beaches (Cheung et al. 1990) or bathing areas in any other part of the world where the maximal aRs were < 5% or 3%.

Microbiological quality standards are intended to protect the health of those consumers who are not already immune or for some other reason resistant to the pathogens from which they are to be protected. For example, they should provide a certain degree of protection not only for the local population under endemic conditions but also for tourists who may have a completely different susceptibility status with respect to a typical pattern of pathogenic organisms to which local residents are already adapted. In addition, standards should also protect the consumers’ health in the situation of epidemics caused by newly introduced pathogens, when even the local population may be extremely susceptible. Therefore, the highly variable cohort susceptibilities that can be observed in epidemiologic studies investigating the risk of acquiring gastroenteritis from recreational water contact should not play a determining role in the setting of standards. Instead, the standards should be based solely on acceptable rates of compliance with guide values that correspond to experimentally and empirically verified NOAELs. In this case, the level of a standard would depend on only one variable, the NOAEL, instead of three: the NOAEL, the cohort susceptibility, and the disease definition. The advantages of choosing guide values in the same order of magnitude of NOAELs are manifold: *a*) the levels of the guide values would be easy to communicate to the public, because the meaning of a NOAEL is easy to understand; *b*) different definitions of gastroenteritis and different cohort susceptibilities in certain epidemiologic studies would not automatically result in different standards; and *c*) the results of individual water samples could be directly compared with the standard (i.e., values below NOAEL vs. values above NOAEL or, e.g., 10 times above NOAEL), whereas such a comparison is not informative if standards are expressed as 95th percentiles of the distribution of microbial concentrations as currently proposed by the European Commission (CEC 2002). We are fully aware that a NOAEL for gastroenteritis is not a natural constant if it is expressed as a concentration of indicator organisms in the water. In an experimental study design, the NOAEL is influenced by the pathogen:indicator ratio, the virulence of the pathogens, the amount of water that is ingested, and, depending on the mathematical method used to determine the NOAEL, possibly the cohort size and the associated power of the statistical analysis. Therefore, the intensity of exposure in experimental studies should be similar to an average bathing activity, and NOAELs determined in experimental studies such as the randomized trials in the United Kingdom (Kay et al. 1994) and in Germany (present results) should be compared with empirical observations before they are used as a basis for standards.

According to the results of the present study and the considerations described above, the following values are suggested as reasonable estimates for NOAELs at an average bathing intensity and as a practicable basis for setting recreational water standards: 100 *E. coli*, 25 intestinal enterococci, 10 somatic coliphages, or 10 *C. perfringens* per 100 mL. These values are well in accordance with empirical observations for the concentration of intestinal enterococci and *E. coli* as demonstrated by the meta-analysis performed by Wade et al. (2003) for the U.S. Centers for Disease Control and Prevention. They concluded that there was evidence that the risk of gastrointestinal illness was considerably lower in studies with indicator densities below the freshwater guidelines proposed by the U.S. Environmental Protection Agency (EPA) for both enterococci (33/100 mL) and *E. coli* (126/100 mL). The results of the randomized trials in German fresh waters are also in accordance with the ratio of roughly 1:4 between the guideline values for enterococci and *E. coli* recommended by the U.S. EPA and are in clear contrast to the ratio of 1:2.5 that is assumed by the European Commission in the current draft directive (CEC 2002).

A novel and practicable monitoring system based on compliance rates with NOAELs (calculation of “time-integrated quality scores”) was recently proposed (Wiedenmann A and Botzenhart K, unpublished data). Current WHO recommendations and the concept used to derive bathing water standards in the European Union should be reconsidered in light of the results and conclusions from this randomized trial.

The results of the present study also demonstrate that *C. perfringens* and somatic coliphages may be alternative fecal indicators that could be used to set standards for fresh water just as well as *E. coli* and enterococci. These results can be of interest in regions such as those with tropical climates, where *E. coli* and enterococci may be less reliable as indicator organisms, because there is some indication that these organisms might multiply in such environments (Fujioka and Byappanahalli 2003).

For the analysis of the trial results reported in this article, we have applied the most conservative concentration–response model consisting of only a NOAEL, the incidence rate of disease below the NOAEL, and the incidence rate of disease above the NOAEL. The modeling of complex concentration–response functions to only few cases of disease may lead to highly speculative results, especially when the models are extrapolated (Kay et al. 2004). We are, however, ready to test any of the various possible modeling approaches (Kay et al. 2004; Wymer and Dufour 2002) or to perform meta-analyses in cooperation with other research groups.

## Supplementary Material

Supplemental material Annexes

## Figures and Tables

**Table 1:** RRs and aRs of bathing above NOAELs versus nonbathing, and aRs of swallowing water versus not swallowing water below and above NOAELs by exposure definition, fecal indicator, and definition of gastroenteritis 1 week after exposure.

Exposure definition^a^	Fecal indicator	Definition of GE	NOAEL (MO/100 mL)	RR, bathing > NOAEL	95% CI	aR (%), bathing > NOAEL	aR (%), SW ≤ NOAEL	aR (%), SW > NOAEL
1	*E. coli*	UK	180	3.55	1.79–7.02	3.6	1.3	3.9
		UK-wf	78	2.51	1.55–4.05	4.3	1.6	2.9
		NL-2	167	1.96	1.32–2.89	5.0	1.0	3.3
	Intestinal enterococci	UK	24	3.2	1.64–6.27	3.1	1.3	3.7
		UK-wf	21	2.67	1.65–4.32	4.7	0.7	3.4
		NL-2	24	1.9	1.30–2.77	4.7	2.8	2.3
	*C. perfringens*	UK	13	3.34	1.72–6.51	3.3	0.4	4.1
		UK-wf	13	2.61	1.60–4.25	4.5	0.9	3.3
		NL-2	13	1.86	1.27–2.73	4.5	0.8	3.5
	Somatic coliphages	UK	150^b^	4.61	2.1–10.11	5.1	1.3	6.7
		UK-wf	10	2.47	1.51–4.04	4.2	1.6	2.8
		NL-2	10	1.77	1.21–2.60	4.0	2.1	2.1
2	*E. coli*	UK	1,453	4.41	2.17–8.96	5.2	1.6	5.3
		UK-wf	—c	—	—	—	—	—
		NL-2	2,163	2.33	1.48–3.67	8.6	0.5	8.6
	Intestinal enterococci	UK	123	3.41	1.74–6.68	3.7	1.1	4.0
		UK-wf	123	2.78	1.71–4.51	5.7	1.9	2.8
		NL-2	145	1.93	1.31–2.84	6.0	3.8	1.6
	*C. perfringens*	UK	38	3.12	1.62–6.03	3.2	−0.4	4.1
		UK-wf	38	2.43	1.50–3.93	4.6	0.8	3.3
		NL-2	36	1.71	1.18–2.47	4.6	0.0	3.2
	Somatic coliphages	UK	330	3.77	1.87–7.61	4.2	1.4	3.2
		UK-wf	50	2.44	1.49–3.98	4.6	1.7	2.7
		NL-2	119	1.76	1.18–2.62	4.9	0.7	3.1

Abbreviations: CI, confidence interval; GE, gastroenteritis; MO, microorganisms; SW, swallowing water.

a Exposure definitions 1 and 2 as explained in “Materials and Methods.”

b Value is probably too high because of a second local minimum of the Pearson’s chi-squared *p*-values.

c Potential NOAEL estimate does not fulfill all criteria of validity (incidence rate of bathers below NOAEL is significantly lower than incidence rate of nonbathers).

**Table 2:** Incidence rates of gastroenteritis (definition GE_UK-wf) in quartile and quintile categories of microbial exposure concentrations for exposure definition 1 (“10 min bathing with at least three head immersions”) 1 week after exposure.

	Quartile UL					Quintile UL				
Parameter	MO/100 mL	Cases	No.	IR (%)	*p*-Value^a^	MO/100 mL	Cases	No.	IR (%)	*p*-Value
Unexposed		26	921	2.8			26	921	2.8	
*E. coli*	72	4	207	1.9	0.47	61	3	166	1.8	0.61
	181	11	212	5.2	0.08	116	6	168	3.6	0.62
	379	14	211	6.6	0.007**	245	10	170	5.9	0.04*
	4,600	17	208	8.2	< 0.001#	445	12	166	7.2	0.004**
						4,600	15	168	8.9	< 0.001#
Intestinal enterococci	14	5	208	2.4	0.74	12	3	167	1.8	0.61
	53	9	212	4.2	0.28	27	5	167	3.0	0.80
	101	14	210	6.7	0.007**	68	15	169	8.9	< 0.001#
	1,190	18	208	8.7	< 0.001#	114	7	168	4.2	0.35
						1,190	16	167	9.6	< 0.001#
*C. perfringens*	9	6	224	2.7	0.91	9	6	224	2.7	0.91
	18	9	202	4.5	0.23	13	4	115	3.5	0.57
	33	18	211	8.5	< 0.001#	22	12	172	7.0	0.006**
	148	13	210	6.2	0.02*	36	14	166	8.4	< 0.001#
						148	10	170	5.9	0.04*
Somatic coliphages	10	7	302	2.3	0.64	10	7	302	2.3	0.64
	35	9	115	7.8	0.01*	11	3	31	9.7	0.06
	142	12	212	5.7	0.04*	85	11	167	6.6	0.01*
	3,598	16	206	7.8	< 0.001#	153	10	171	5.8	0.04*
						3,598	13	164	7.9	0.001**

Abbreviations: IR, incidence rate; MO, microorganisms; UL, upper range limit.

a The *p*-value was calculated by Pearson’s chi-square test or Fisher’s exact test that compared the number of cases among the bathers who were exposed in one of the given categories of fecal indicator concentrations with the number of cases among the unexposed participants (nonbathers). Fisher’s exact test results were used when an expected cell value was < 5.

*p < 0.05;

**p < 0.01;

# *p* < 0.001.
