# Supplementary material for: A Randomized Controlled Trial Assessing Infectious Disease Risks from Bathing in Fresh Recreational Waters in Relation to the Concentration of Escherichia coli, Intestinal Enterococci, Clostridium perfringens, and Somatic Coliphages
Source: Environ Health Perspect. 2005 Sep 29;114(2):228–36. doi: 10.1289/ehp.8115 (PMC1367836; doi:10.1289/ehp.8115)
Supplement: Supplemental material Annexes [file ehp0114-000228s1.pdf]

Supplemental Data, Annex 1. Geographical site characteristics and sources of fecal contamination

Site	Geographical characteristics	Probable or possible sources of fecal contamination
1	Ground water lake, former gravel mining area, in rural environment with adjacent conservation areas (Southwest of Germany, Baden-Württemberg)	Sparse natural populations of water fowl, mainly swans, ducks and great crested grebes, accidental fecal release from bathers
2	Germany's biggest natural inland lake in urban environment with the Rhine River running through and many smaller rivers and streams running into it (Southwest of Germany, Baden-Württemberg)	Discharge from tertiary treated sewage, sporadic sewage treatment plant and combined sewer overflows, sporadic agricultural run-off, illegal discharge from boats, urban and natural populations of water fowl, mainly ducks and coots, accidental fecal release from bathers
3	Ground water lake in urban environment (Northeast of Germany, Berlin)	Urban and natural populations of water fowl, mainly ducks and grey herons, accidental fecal release from bathers
4	Blind arm of a stream in urban environment (North of Germany, Schleswig-Holstein)	Combined sewer overflows and urban run-off after a heavy rain fall the day before, urban and natural populations of water fowl mainly ducks, gulls and swans, accidental fecal release from bathers
5	Ground water lake in urban environment (Southeast of Germany, Bavaria)	High overpopulation with ducks, geese and gulls, accidental fecal release from bathers

Supplemental Data, Annex 2. Median concentrations and concentration ranges of the microbiological parameters.

Parameter	Median concentration (MO/100ml)	Range [LL; UL]	Water samples n	Method
Escherichia coli	136	[4.7; 5344] [a]	421	ISO 9308-3 [b]
Inestinal enterococci	37	[3.0; 1504] [a]	421	ISO 7899-1 [c]
Clostridium perfringens	18	[9; 260] [a]	423	CEU 1998 [d]
Somatic coliphages	20	[10; 3780] [a]	420	ISO 10705-2 [e]
Aeromonads	8200	[600; 31400]	420	Schulze 1996 [f]
Pseudomonas aeruginosa	10	[10; 100] [a]	385	DIN EN 12780 [g]

Abbreviations: MO/100mL, microorganisms per 100 mL; n, number; LL, lower limit; UL, upper limit. [a] LL is also the lower method detection limit. [b] MUG hydrolysis, microtiter plate method, 3 plates per sample, 200 µl per well, 192 wells with a 1:2 dilution, 96 wells with a 1:10 dilution. [c] MUD hydrolysis and formazan formation, 3 plates per sample, 200 µl per well, 288 wells with a 1:2 dilution. [d] Membrane filtration, 10 mL and 1 mL on m-CP agar. [e] Double agar layer method, 10 x 1 ml, sample pre-filtration with beef-extract-saturated 0.45 µm membrane filters. [f] Membrane filtration, 10 mL and 1 mL on ampicillin-dextrine agar. [g] Membrane filtration, 10 mL on cetrimide agar, only pyocyanine-positive colonies were counted .

Supplemental Data, Annex 3. Correlation between microbiological parameters, sorted by correlation coefficient, after exclusion of censored values i. e. values below detection limit.

Parameter X	Parameter Y	n	Intercept [a]	Slope [b]	Correlation coefficient
Escherichia coli	Inestinal enterococci	391	-0.270	0.836	0.79
Escherichia coli	Somatic coliphages	243	-1.112	1.243	0.78
Escherichia coli	*Clostridium perfringens*	285	-0.907	0.537	0.57
Inestinal enterococci	*Clostridium perfringens*	278	-0.714	0.631	0.51
Clostridium perfringens	Somatic coliphages	209	1.137	2.115	0.50
Inestinal enterococci	Somatic coliphages	241	-0.802	1.525	0.41
Inestinal enterococci	*Pseudomonas aeruginosa*	50	-1.370	0.841	0.31
Escherichia coli	*Pseudomonas aeruginosa*	50	-1.685	0.640	0.29
Somatic coliphages	*Pseudomonas aeruginosa*	49	-1.099	0.483	0.28
Escherichia coli	Aeromonads	405	2.779	0.492	0.27
Somatic coliphages	Aeromonads	242	2.760	0.529	0.24
Clostridium perfringens	Aeromonads	285	3.473	1.030	0.23
Clostridium perfringens	*Pseudomonas aeruginosa*	47	-0.252	0.795	0.17
Inestinal enterococci	Aeromonads	399	2.871	0.617	0.11
Aeromonads	*Pseudomonas aeruginosa*	48	-4.789	1.211	0.07

Abbreviations: n, Number of water samples.
[a] Intercept in the orthogonal fit line equation: log10(parameter Y) = intercept + slope x log10(parameter X).
[b] slope of the orthogonal fit line

Supplemental Data, Annex 4. Crude incidence rates, attributable risks and relative risks of bathers vs. non-bathers one and three weeks after exposure.

	Non-bathers				Bathers				Bathers vs. non-bathers					
	Incidence rate (%) [a]		n		Incidence rate (%)		n		*p*-Value [b]		aR [c]		RR [d]	
Disease	1 w	3 w	1 w	3 w	1 w	3 w	1 w	3 w	1 w	3 w	1 w	3 w	1 w	3 w
AFRI	1.1	2.2	810	802	0.9	1.7	759	747	0.71	0.48	-0.2	-0.5	0.8	0.8
CC	13.6	20.4	804	793	15.4	23.1	754	741	0.31	0.21	1.8	2.6	1.1	1.1
EAR-I	0.7	1.3	1003	1001	1.6	2.2	950	942	0.065	0.12	0.9	0.9	2.3	1.7
EYE-I	1.6	2.5	999	997	1.7	2.7	944	938	0.87	0.83	0.1	0.2	1.1	1.1
GE_UK	1.4	4.8	920	902	3.3	6.6	837	818	0.0074 **	0.10	1.9	1.8	2.4	1.4
GE_UK-wf	2.8	7.1	921	907	5.4	9.0	847	831	0.0056 **	0.13	2.6	2.0	1.9	1.3
GE_NL-2	5.2	11.1	921	908	7.4	13.0	846	823	0.053 (*)	0.23	2.2	1.9	1.4	1.2
SKIN-I	2.8	4.3	957	947	9.3	13.0	880	869	<0.0001 ***	<0.0001 ***	6.5	8.7	3.3	3.0
UTI	0.4	1.1	1002	999	0.2	0.4	944	939	0.46	0.090	-0.2	-0.7	0.5	0.4

Abbreviations: n, number of participants; aR, attributable risk; RR, relative risk; 1 w, within one week after exposure; 3 w, within three weeks after exposure; AFRI, acute febrile respiratory infections: (fever and sore throat, sphagitis) or (fever and pain in the chest) or (fever and dry cough) or (fever and productive cough) or (fever and breathing difficulties) or (fever and runny nose, coryza); CC, common cold: sore throat, sphagitis or dry cough or productive cough or runny nose, coryza; EAR-I, ear inflammation: inflammation, suppuration, pain in the ears; EYE-I, eye inflammation: inflammation, suppuration of the eyes, red painful eyes; GE_UK, gastroenteritis definition UK: (diarrhoea and 3 or more bowel movements per day) or vomiting or (nausea and fever) or (indigestion and fever); GE_UK-wf, gastroenteritis definition UK-wf: diarrhoea or vomiting or (nausea and fever) or (indigestion and fever); GE_NL-2, gastroenteritis definition NL-2: diarrhoea or vomiting or nausea or stomach pains; SKIN-I, skin inflammation or cutireations: exanthema, skin eruption or skin irritation, itching; UTI: inflammation of the urinary bladder, urinary tract, urinary tract infection. [a] Incidence rate (%) is the number of individuals with onset of disease within one or three weeks after exposure per 100 participants in the group of non-bathers or bathers. [b] *p*-Value in a Pearson's Chi Square test comparing the incidence rates of bathers and non-bathers. [c] Attributable risk is the incidence rate (%) of bathers minus the incidence rate (%) of non-bathers. [d] Relative risk is the incidence rate (%) of bathers divided by the incidence rate (%) of non-bathers. * *p*-Value <0.05. ** *p*-Value <0.01. *** *p*-Value <0.001. (*) borderline significance.

Supplemental Data, Annex 5. Univariate screening for potential confounding variables

**Variables**		**Disease definitions**				
CODE [a]	Explanation (result categories)	CC	UK	UK-wf	NL-2	SKIN
**Ambient conditions**						
LOCATION	Study location (1, 2, 3, 4, 5)	*	*	-	-	*
WEATHER_TEMP	Weather conditions (cool or warm)	*	-	-	-	*
**Interviewer effect**						
INR3	Number of the interviewer in the 3rd interview (number)	-	-	-	-	-
**Age**						
AGE	Age (in years)	-	-	-	-	-
AGE-GROUP	Age (grouped by 10-years intervals)	*	-	-	-	-
AGE-GROUP_2	Age (grouped by 10-year intervals and 60+)	*	-	-	-	-
**Gender**						
SEX1	Gender (male or female)	-	-	-	-	-
**Socio-economic status**						
DEGREE1	Degree (none, primary school, non-classical secondary school, secondary school 12th class, secondary school 13th class, non-classical university, classical university, other)	*	-	-	-	-
DEGREE1_2	Degree (none, primary school, non-classical secondary school, secondary school, university)	*	-	-	-	*

Supplemental Data, Annex 5. (continued)

**Variables**		**Disease definitions**				
CODE [a]	Explanation (result categories)	CC	UK	UK-wf	NL-2	SKIN
**Socio-economic status**						
OCCUP1	Occupation (unemployed, child or pupil, student, household, practicing a profession, retired)	*	-	-	-	-
OCCUP1_2	Occupation (unemployed, child or pupil, student, household, practicing a profession)	*	-	-	-	-
**Household**						
HHMEMB1	Number of household members (number)	-	-	-	-	-
HHMEMB1_2	Number of household members (1, 2, 3, 4, 5, >5)	-	-	-	-	-
HHCHILD1	Number of children < 5 years of age living in household	-	-	-	-	-
HHCHILD1_2	Number of children < 5 years of age living in household (none, 1, >1)	-	*	-	*	-
HHCHILD1_3	Children < 5 years living in household (yes, no)	nd	*	*	*	nd
HHVOL2	Other household members participating in the study (yes, no)	-	-	-	-	-
HHCOLD1	Household member with common cold in 2 weeks before 1st interview (yes, no)	*	-	-	-	nd
HHTHRO1	Household member with sore throat in 2 weeks before 1st interview (yes, no)	-	nd	nd	nd	nd
HHLUNG1	Household member with lung infection in 2 weeks before 1st interview (yes, no)	-	nd	nd	nd	nd
HHFEV1	Household member with fever in 2 weeks before 1st interview (yes, no)	-	-	-	-	-
HHNAUS1	Household member with nausea in 2 weeks before 1st interview (yes, no)	nd	-	-	-	nd
HHVOM1	Household member with vomiting in 2 weeks before 1st interview (yes, no)	nd	-	-	-	nd
HHDIAR1	Household member with diarrhea in 2 weeks before 1st interview (yes, no)	nd	-	-	-	nd
HHSKIN1	Household member with skin ailments in 2 weeks before 1st interview (yes, no)	nd	nd	nd	nd	-
**Chronic symptoms**						
CCHEST1	Chronic chest ailments (yes, no)	-	nd	nd	nd	nd
CEAR1	Chronic ear ailments (yes, no)	-	nd	nd	nd	nd
CHAY1	Chronic hay fever (yes, no)	*	nd	nd	nd	-

Supplemental Data, Annex 5. (continued)

Variables		Disease definitions				
CODE [a]	Explanation (result categories)	CC	UK	UK-wf	NL-2	SKIN
CSTOM1	Chronic stomach ailments (yes, no)	nd	-	*	*	nd
CGUT1	Chronic gut ailments (yes, no)	nd	-	-	-	nd
ILLDIARFR1	Normal frequency of diarrhea	nd	*	*	*	nd
	(never, hardly ever: 1-2 per year, often: 1-2 per month)					
TRAVSICK4	Proneness to motion sickness (always, often, hardly ever, never)	nd	-	-	*	nd
CLIV1	Chronic liver ailments (yes, no)	nd	-	-	-	-
CSKIN1	Chronic skin ailments (yes, no)	nd	nd	nd	nd	*
CREN1	Chronic renal ailments (yes, no)	nd	nd	nd	nd	-
CNEUR1	Chronic neural ailments (yes, no)	nd	nd	nd	nd	-
CDIAB1	Chronic diabetes (yes, no)	-	-	-	-	-
ILL6MON1	Any severe illness in 6 months before 1st interview (yes, no)	-	-	-	-	-
ILLHOSP1	Stay in hospital in 6 months before 1st interview (yes, no)	-	-	-	-	-
**Symptoms or ailments**						
SYFEV1	Fever in three weeks before 1st interview (yes, no)	*	-	-	-	-
SYFEV2	Fever between 1st and 2nd interview (yes, no)	nd	nd	nd	nd	-
SYHEAD1	Headache in three weeks before 1st interview (yes, no)	*	nd	nd	nd	nd
SYNOSE1	Runny nose in three weeks before 1st interview (yes, no)	-	nd	nd	nd	nd
SYDCOUGH1	Dry cough in three weeks before 1st interview (yes, no)	-	nd	nd	nd	nd
SYPCOUGH1	Productive cough in three weeks before 1st interview (yes, no)	-	nd	nd	nd	nd
SYTHRO1	Sore throat in three weeks before 1st interview (yes, no)	-	nd	nd	nd	nd
SYBREATH1	Breathing problems in three weeks before 1st interview (yes, no)	-	nd	nd	nd	nd
SYCHEST1	Pain in the chest in three weeks before 1st interview (yes, no)	-	nd	nd	nd	nd
SYJOINT1	Pain in the joints in three weeks before 1st interview (yes, no)	-	nd	nd	nd	nd
SYEAR1	Ear inflammation in three weeks before 1st interview (yes, no)	-	nd	nd	nd	nd
SYAPP1	Loss of appetite in three weeks before 1st interview (yes, no)	-	-	-	-	nd

Supplemental Data, Annex 5. (continued)

**Variables**		**Disease definitions**				
CODE [a]	Explanation (result categories)	CC	UK	UK-wf	NL-2	SKIN
SYINDIG1	Indigestion in three weeks before 1st interview (yes, no)	nd	-	-	-	nd
SYNAUS1	Nausea in three weeks before 1st interview (yes, no)	nd	-	-	-	nd
SYVOM1	Vomiting in three weeks before 1st interview (yes, no)	nd	-	-	-	nd
SYSTOM1	Stomach pains or cramps in three weeks before 1st interview (yes, no)	nd	-	-	*	nd
SYLOOSEB1	Loose bowel motions in three weeks before 1st interview (yes, no)	nd	*	*	*	nd
SYDIAR1	Diarrhea in three weeks before 1st interview (yes, no)	nd	*	*	*	nd
SYANY_GI	Any gastrointestinal disorders in three weeks before 1st interview (yes, no)	nd	-	-	*	nd
SYSKIN1	Skin ailments in three weeks before 1st interview (yes, no)	nd	nd	nd	nd	-
SYITCH1	Itching in three weeks before 1st interview (yes, no)	nd	nd	nd	nd	*
SYPIN1	Needles and pins in three weeks before 1st interview (yes, no)	nd	nd	nd	nd	-
SYSKINUL1	Skin ulcer in three weeks before 1st interview (yes, no)	nd	nd	nd	nd	-
**Medicines**						
MED1	Consumption of prescription drugs in 4 weeks before 1st interview (yes, no)	-	-	-	-	-
MEDANTI1	Antibiotics in 4 weeks before 1st interview (yes, no)	-	-	-	-	-
MEDCORT1	Steroids in 4 weeks before 1st interview (yes, no)	-	-	-	-	-
MEDLAX1	Laxatives in 4 weeks before 1st interview (yes, no)	nd	-	-	-	nd
MEDSTOM1	Stomach remedies in 4 weeks before 1st interview (yes, no)	nd	-	-	-	nd
**Food**						
FRMEAT2	Consumption of raw meat in two or three days before exposure (yes, no)	nd	-	-	-	nd
nd FRMEAT3	Consumption of raw meat in the week after exposure (yes, no)	nd	-	-	-	nd
FRMILK2	Consumption of raw milk in two or three days before exposure (yes, no)	nd	-	-	-	nd
FRMILK3	Consumption of raw milk in the week after exposure (yes, no)	nd	-	-	-	nd
FRCHEESE2	Consumption of raw milk cheese in two or three days before exposure (yes, no)	nd	-	-	-	nd
FRCHEESE3	Consumption of raw milk cheese in the week after exposure (yes, no)	nd	-	-	-	nd

Supplemental Data, Annex 5. (continued)

**Variables**		**Disease definitions**				
CODE [a]	Explanation (result categories)	CC	UK	UK-wf	NL-2	SKIN
FEGG2	Consumption of scrambled eggs, omelets in two or three days before exposure (yes, no)	nd	-	-	-	nd
FEGG3	Consumption of scrambled eggs, omelets in the week after exposure (yes, no)	nd	-	-	-	nd
FMAYO2	Consumption of self made mayonnaise in two or three days before exposure (yes, no)	nd	-	-	-	nd
FMAYO3	Consumption of self made mayonnaise in the week after exposure (yes, no)	nd	-	-	-	nd
FTIRA2	Consumption of tiramisu in two or three days before exposure (yes, no)	nd	-	-	-	nd
FTIRA3	Consumption of tiramisu in the week after exposure (yes, no)	nd	-	-	-	nd
FICE2	Consumption of ice cream in two or three days before exposure (yes, no)	nd	-	-	-	nd
FICE3	Consumption of ice cream in the week after exposure (yes, no)	nd	-	-	-	nd
FCHICK2	Consumption of chicken meat or poultry in two or three days before exposure (yes, no)	nd	-	-	-	nd
FCHICK3	Consumption of chicken meat or poultry in the week after exposure (yes, no)	nd	-	-	-	nd
FFASTF2	Consumption of fast-food in two or three days before exposure (yes, no)	nd	-	-	-	nd
FFASTF3	Consumption of fast-food in the week after exposure (yes, no)	nd	-	-	-	nd
FBURGER2	Consumption of hamburgers in two or three days before exposure (yes, no)	nd	-	-	-	nd
FBURGER3	Consumption of hamburgers in the week after exposure (yes, no)	nd	-	-	-	nd
FHOTDOG2	Consumption of hot digs in two or three days before exposure (yes, no)	nd	-	*	-	nd
FHOTDOG3	Consumption of hot dogs in the week after exposure (yes, no)	nd	-	-	-	nd
FSAUSAGE2	Consumption of grilled sausages in two or three days before exposure (yes, no)	nd	-	-	-	nd
FSAUSAGE3	Consumption of grilled sausages in the week after exposure (yes, no)	nd	-	-	-	nd
FSANDW2	Consumption of bought sandwiches in two or three days before exposure (yes, no)	nd	-	-	-	nd
FSANDW3	Consumption of bought sandwiches in the week after exposure (yes, no)	nd	*	-	*	nd
FSALAD2	Consumption of salads in two or three days before exposure (yes, no)	nd	-	-	-	nd
FSALAD3	Consumption of salads in the week after exposure (yes, no)	nd	-	-	-	nd

Supplemental Data, Annex 5. (continued)

Variables		Disease definitions				
CODE [a]	Explanation (result categories)	CC	UK	UK-wf	NL-2	SKIN
FSEAF2	Consumption of sea food in two or three days before exposure (yes, no)	nd	-	-	-	nd
FSEAF3	Consumption of sea food in the week after exposure (yes, no)	nd	-	-	-	nd
FBBQ2	Participation in a barbecuing party in two or three days before exposure (yes, no)	nd	-	-	-	nd
FBBQ3	Participation in a barbecuing party in the week after exposure (yes, no)	nd	-	-	-	nd
**Alcohol**						
ALC1	Consumption of alcohol (yes, no)	-	-	-	-	*
ALC7D1	Amount of alcohol consumed in the week before 1st interview (number of units (1 unit = 0.5 l beer, 0.25 l wine, 0.02 l spirits))	-	-	-	-	-
ALC7D1_2	Amount of alcohol consumed in the week before 1st interview (0, 1-5, 6-10, >10 units (1 unit = 0.5 l beer, 0.25 l wine, 0.02 l spirits))	-	-	-	-	-
ALCUS1	Was this the normal amount of alcohol (yes, no)	-	-	-	-	-
**Tobacco**						
SMOKE1	Smoking (yes, yes only pipe, no)	-	-	-	-	-
SMOKE1_2	Smoking (yes, no)	-	-	-	-	-
SMOKENR1	Number of cigarettes per day (mean number)	-	-	-	-	-
SMOKENR1_2	Number of cigarettes per day (0, 1-5, 6-20, >20)	-	-	-	-	-
**Leisure activities**						
LEPARTY1	Normal frequency of party or disco visits per month (never, sometimes: 1-3, often: >3)	*	-	-	-	-
LEPUB1	Normal frequency of pub visits per month (never, sometimes: 1-3, often: >3)	*	-	*	-	*
LESPORT1	Normal frequency of sports activities per month (never, sometimes: 1-3, often: >3)	*	-	-	*	-

Supplemental Data, Annex 5. (continued)

**Variables**		**Disease definitions**				
CODE [a]	Explanation (result categories)	CC	UK	UK-wf	NL-2	SKIN
LECIN1	Normal frequency of cinema visits per month (never, sometimes: 1-3, often: >3)	-	nd	nd	nd	nd
LEOTHER1	Normal frequency of other activities involving contact to large groups of people (never, sometimes: 1-3, often: >3)	-	nd	nd	nd	nd
**Travel history**						
TRAVEL1	Overnight stays outside household in 4 weeks before 1st interview (yes, no)	*	-	-	-	-
TRAVPL1	Traveling abroad in 4 weeks before 1st interview (yes, no)	-	-	-	-	-
**Water related**						
BP_WATER	Exposed and swallowed water during trial (no, tea-spoon, table-spoon, cup)	-	-	-	-	nd
BP_Water_B-nein_B-ja	Exposed and swallowed water during trial (yes, no)	-	-	*	-	nd
BP_Water_NB=0_B=1-4	Swallowed water during trial (unexposed or exposed and no, tea-spoon, table-spoon, cup)	-	*	*	-	nd
BP_Water_NB_B-n_B-j	Swallowed water during trial (unexposed, exposed and no, exposed and yes)	-	*	*	*	nd
BATH1	Normal frequency of bathing in natural recreational waters (fresh or sea water) per month during summer (never; sometimes: 1-3; often: >3)	-	-	-	-	-
BATH2	Swimming or bathing in the two or three days between 1st and 2nd interview (yes, no)	-	-	-	-	-
BATH3	Additional swimming or bathing in the week after the trial day (yes, no)	-	-	-	-	-
BATHFR1	Frequency of bathing in 4 weeks before 1st interview	-	-	-	-	-
BATHFR1_2	Frequency of bathing in 4 weeks before 1st interview (0, 1-5, >5)	-	-	-	-	-
HBEACH1	Normal frequency of going to a beach without entering the water per month (never, sometimes: 1-3, often: >3)	*	-	-	-	-

Supplemental Data, Annex 5. (continued)

Variables		Disease definitions				
CODE [a]	Explanation (result categories)	CC	UK	UK-wf	NL-2	SKIN
HBEACHFR1	Number of beach visits without entering the water in the 4 weeks before 1st interview (number)	-	-	-	-	-
HBEACHFR1_2	Number of beach visits without entering the water in 4 weeks before 1st interview (0, 1-5, >5)	-	-	-	-	-
HBEACHFR1_3	Beach visits without entering the water in 4 weeks before 1st interview (yes, no)	-	nd	nd	nd	nd
HPUPOOL1	Normal frequency of using a public pool per month (never, sometimes: 1-3, often: >3)	*	-	-	-	-
HPRPOOL1	Normal frequency of using a private pool per month during summer (never; sometimes: 1-3; often: >3)	-	-	-	-	-
HPRPOOL1_2	Utilization of private pools (yes, no)	*	-	-	-	-
HLEPARK1	Visit to a leisure park with water activities in 4 weeks before 1st interview (yes, no)	-	-	-	-	*
BASWIMDIS1	Maximal swimming distance (number of meters)	-	-	-	-	-
BASWIMDIS1_2	Maximal swimming distance (-50, >50 m)	-	-	-	-	-
BASWIMDUR1	Maximal swimming duration (number of minutes)	-	-	-	-	-
BASWIMDUR1_2	Maximal swimming duration (10 min, 20 min, 30 min, 1 h, 2 h)	-	-	-	-	-
WASURF1	Normal frequency of surfing during summer (never, sometimes: 1-3, often: >3)	*	-	-	-	-
WAPAD1	Normal frequency of paddling during summer (never, sometimes: 1-3, often: >3)	-	-	-	-	-
WACANOE1	Normal frequency of canoeing during summer (never, sometimes: 1-3, often: >3)	*	-	-	-	-
WAMOTOR1	Normal frequency of motorboating during summer (never, once or more)	*	-	-	-	-
WADIV1	Normal frequency of diving during summer (never, once or more)	*	-	-	-	-
WAFISH1	Normal frequency of fishing during summer (never, once or more)	-	-	-	-	-

Supplemental Data, Annex 5. (continued)

Variables		Disease definitions				
CODE [a]	Explanation (result categories)	CC	UK	UK-wf	NL-2	SKIN
**Risk perception**						
WADA4	Are water related activities considered to be dangerous (yes, no)	-	-	-	-	-
BBILL4	Ever gone to a beach while feeling ill (yes, no)	*	-	-	-	-
BBILLWAT4	Did feeling ill prevent from entering the water (yes, no)	-	-	-	-	-
WQNBBEACH4	Ever refused to go bathing because beach was too dirty (yes, no)	-	*	*	*	-
WQNBWATER4	Ever refused to go bathing because water was too dirty (yes, no)	-	-	*	-	-
WQNBWAVES4	Ever refused to go bathing because waves were too rough (yes, no)	-	-	-	-	-
WQNBHRISK4	Ever refused to go bathing because of fear to become ill (yes, no)	-	-	-	-	*
SUNBFR4	Frequency of sunburns after beach visits (always, often, hardly ever, never)	nd	nd	nd	nd	-
SUNBMED4	Are sunburns treated (always, often, hardly ever, never)	nd	nd	nd	nd	-
**Information**						
IRECRUIT4	Initial source of information about this study (partner, recruitment team, television, newspaper, other)	-	-	-	-	-
IMEDIA4	Having seen or heard news about this study (yes, no)	-	-	-	-	-
INEWSPAP4	Regular reading of a newspaper (yes, no)	-	-	-	-	-
IENVORG4	Member of an environmental organisation (yes, no)	-	-	-	-	-
WQCONTR4	Informed about the control of beaches in Germany (yes, no)	*	-	-	-	-
WQCONTRPN4	Quality of information on control of beaches in Germany (positive or negative)	*	-	-	-	*
WQCONTRWO4	Worried about information on the control of beaches in Germany (no, a bit, very much)	-	-	-	-	-
WQCONTRWO4_2	Worried about information on the control of beaches in Germany (yes, no)					
WQCLEAN4	Information on the cleanliness of recreational waters in Germany (yes, no)	*	*	-	*	-
WQCLEANPN4	Quality of information on cleanliness of German recreational waters (positive or negative)	*	-	-	-	-

Supplemental Data, Annex 5. (continued)

Abbreviations: CC, common cold; UK, Gastroenteritis definition UK; UK-wf, Gastroenteritis definition UK-wf; NL-2, Gastroenteritis definition NL-2; nd, not done. [a] CODE is the variable code in the computer data set. * *p*-Value for Pearson's Chi Square test < 0.05. - *p*-Value for Pearson's Chi Square test $\geq$ 0.05.

Supplemental Data, Annex 6. Analysis of potential NOAELs and potential confounding variables by multiple logistic regression (Effect Likelihood Ratio Tests) for exposure definition 1 ("10 minutes bathing; three or more head immersions").

**Disease** Indicator	Potential NOAEL	*p*-Value [a]	Better predictor [b]	Status	Confounding or interacting variable	*p*-Value [c]
**Acute febrile respiratory infections**						
EC	---					
IE	---					
CP	---					
SOMCP	---					
AE	---					
PA	---					
**Common cold**						
EC	261	0.0007		Confounded by	Quality of information on control of beaches in Germany	0.0067
				Confounded by	Quality of information on cleanliness of German recreational waters	0.0184
IE	132	0.0010		Confounded by	Quality of information on control of beaches in Germany	0.0073
				Interaction with	Headache in three weeks before 1st interview	0.0100
				Interaction with	Ever gone to a beach while feeling ill	0.0417
CP	30 [d]	0.0220		Confounded by	Study location	0.0014
				Confounded by	Weather conditions	0.0001
				Confounded by	Age grouped by 10-years intervals	0.0000
				Confounded by	Age grouped by 10-year intervals and 60+	0.0000
				Confounded by	Degree	0.0037
				Confounded by	Degree (definition 2)	0.0013
				Confounded by	Occupation	0.0005
				Confounded by	Occupation (definition 2))	0.0002

Supplemental Data, Annex 6. (continued).

**Disease** Indicator	Potential NOAEL	*p*-Value [a]	Better predictor [b]	Status	Confounding or interacting variable	*p*-Value [c]
				Confounded by	Quality of information on control of beaches in Germany	0.0114
				Confounded by	Quality of information on control of beaches in Germany	0.0190
				Interaction with	Ever gone to a beach while feeling ill	0.0006
SOMCP	128	0.0012		Confounded by	Study location	0.0125
				Confounded by	Weather conditions	0.0007
				Confounded by	Quality of information on control of beaches in Germany	0.0082
				Confounded by	Quality of information on control of beaches in Germany	0.0195
				Interaction with	Overnight stays outside household in 4 weeks before 1st interview	0.0014
AE	---					
PA	---					
**Ear inflammation**						
IE	---					
CP	---					
SOMCP	---					
AE	---					
PA	---					
**Eye inflammation**						
EC	---					
IE	---					
CP	---					
SOMCP	---					
AE	---					
PA	---					

Supplemental Data, Annex 6. (continued).

**Disease** Indicator	Potential NOAEL	*p*-Value [a]	Better predictor [b]	Status	Confounding or interacting variable	*p*-Value [c]
**Gastroenteritis (Definition UK)**						
EC	180	0.0086		o.k.		
IE	24	0.0220		o.k.		
CP	13	0.0058		o.k.		
SOMCP	150	0.0080	CP, EC, EN	o.k. / Interaction with	Loose bowel motions in three weeks before 1st interview	0.0354
AE	---					
PA	---					
**Gastroenteritis (Definition UK-wf)**						
EC	78	0.0025		o.k.		
IE	21	0.0006		o.k.		
CP	13	0.0019	EN	o.k.		
SOMCP	10	0.0035		o.k. / Interaction with	Loose bowel motions in three weeks before 1st interview	0.0173
AE	---					
PA	---					
**Gastroenteritis (Definition NL-2)**						
EC	167	0.0019		o.k.		
IE	24	0.0010		o.k. / Interaction with	Stomach pains or cramps in three weeks before 1st interview	0.0081
				o.k. / Interaction with	Any gastrointestinal disorders in three weeks before 1st interview	0.0446
CP	13	0.0018		o.k. / Interaction with	Stomach pains or cramps in three weeks before 1st interview	0.0089
SOMCP	10	0.0025		o.k. / Interaction with	Stomach pains or cramps in three weeks before 1st interview	0.0104
				o.k. / Interaction with	Any gastrointestinal disorders	

Supplemental Data, Annex 6. (continued).

**Disease** Indicator	Potential NOAEL	*p*-Value [a]	Better predictor [b]	Status	Confounding or interacting variable	*p*-Value [c]
					in three weeks before 1st interview	0.0172
AE	---					
PA	---					
**Skin inflammation, cutireaction**						
EC	---					
IE	---					
CP	---					
SOMCP	---					
AE	5622	0.0006		o.k. / Interaction with	Weather conditions	0.0022
				o.k. / Interaction with	Chronic skin ailments	0.0000
				o.k. / Interaction with	Itching in three weeks before 1st interview	0.0012
				o.k. / Interaction with	Consumption of alcohol	0.0031
PA	---					
**Urinary tract infection**						
EC	---					
IE	---					
CP	---					
SOMCP	---					
AE	---					
PA	---					

Supplemental Data, Annex 6. (continued).

Abbreviations: EC, *Escherichia coli*; IE, intestinal enterococci; CP, *Clostridium perfringens*; SOMCP, somatic coliphages; AE, aeromonads; PA, *Pseudomonas aeruginosa*; NOAEL, No Observed Adverse Effect Level (Microorganisms/100 mL); o.k., not confounded by any of the tested potential confounding variables; [a] Chi Square *p*-Value in a Pearson's Chi Square test comparing the incidence rates of disease below and above the potential NOAEL. [b] Better predictors are indicator organisms which are significantly better predictors ($p<0.05$) of the outcome (disease) in an Effect Likelihood Ratio Test modeling the disease as response variable and the NOAELs of two indicator organisms as model effects. [c] Chi Square *p*-value for the potential confounding or interacting variable in an Effect Likelihood Ratio Test modeling the disease as response variable and the potential NOAEL and the potential confounding variable as model effects. [d] rounded. --- No concentration fulfilling the validity criteria for a potential NOAEL.

Supplemental Data, Annex 7. Analysis of potential NOAELs and potential confounding variables by multiple logistic regression (Effect Likelihood Ratio Tests) for exposure definition 2 ("Single head immersion").

**Disease** Indicator	Potential NOAEL	*p*-Value [a]	Better predictor [b]	Status	Confounding or interacting variable	*p*-Value [c]
**Acute febrile respiratory infections**						
EC	---					
IE	---					
CP	---					
SOMCP	---					
AE	---					
PA	---					
**Common cold**						
EC	936	0.0130		Confounded by	Study location	0.0019
				Confounded by	Weather conditions	0.0001
				Confounded by	Age grouped by 10-years intervals	0.0000
				Confounded by	Age grouped by 10-year intervals and 60+	0.0000
				Confounded by	Degree	0.0058
				Confounded by	Degree (definition 2)	0.0023
				Confounded by	Occupation	0.0011
				Confounded by	Normal frequency of sports activities per month	0.0174
				Confounded by	Quality of information on control of beaches in Germany	0.0079
				Confounded by	Quality of information on control of beaches in Germany	0.0211
				Interaction with	Ever gone to a beach while feeling ill	0.0091
IE	---					
CP	---					

Supplemental Data, Annex 7. (continued).

**Disease** Indicator	Potential NOAEL	*p*-Value [a]	Better predictor [b]	Status	Confounding or interacting variable	*p*-Value [c]
SOMCP	248	0.0052		Confounded by	Study location	0.0068
				Confounded by	Weather conditions	0.0005
				Confounded by	Degree	0.0054
				Confounded by	Degree (definition 2)	0.0021
				Confounded by	Quality of information on control of beaches in Germany	0.0076
				Confounded by	Quality of information on control of beaches in Germany	0.0226
				Interaction with	Overnight stays outside household in 4 weeks before 1st interview	0.0188
				Interaction with	Normal frequency of diving during summer	0.0208
AE	---					
PA	40 [d]	0.0500		Confounded by	Study location	0.0012
				Confounded by	Weather conditions	0.0001
				Confounded by	Age grouped by 10-years intervals	0.0000
				Confounded by	Age grouped by 10-year intervals and 60+	0.0000
				Confounded by	Occupation	0.0005
				Confounded by	Occupation (definition 2)	0.0002
				Confounded by	Household member with common cold in 2 weeks before 1st interview	0.0170
				Confounded by	Normal frequency of canoeing during summer	0.0237
				Confounded by	Normal frequency of using a private pool per month during summer	0.0132
				Confounded by	Quality of information on control of beaches in Germany	0.0099
				Confounded by	Quality of information on control of beaches in Germany	0.0136
				Interaction with	Degree	0.0441

Supplemental Data, Annex 7. (continued).

**Disease** Indicator	Potential NOAEL	*p*-Value [a]	Better predictor [b]	Status	Confounding or interacting variable	*p*-Value [c]
				Interaction with	Degree (definition 2)	0.0003
				Interaction with	Ever gone to a beach while feeling ill	0.0084
				Interaction with	Normal frequency of surfing during summer	0.0076
**Ear inflammation**						
EC	---					
IE	---					
CP	---					
SOMCP	---					
AE	---					
PA	---			-		
**Eye inflammation**						
EC	---					
IE	---					
CP	---					
SOMCP	---					
AE	---					
PA	---					
**Gastroenteritis (Definition UK)**						
EC	1453	0.0015		o.k.		
IE	123	0.0075		o.k.		
CP	38	0.0080	EC	o.k. / Interaction with	Loose bowel motions in three weeks before 1st interview	0.0354
SOMCP	330	0.0058	EC	o.k.		
AE	---					
PA	---					

Supplemental Data, Annex 7. (continued).

**Disease** Indicator	Potential NOAEL	*p*-Value [a]	Better predictor [b]	Status	Confounding or interacting variable	*p*-Value [c]
**Gastroenteritis (Definition UK-wf)**						
EC	---					
IE	123	0.0005		o.k.		
CP	38	0.0043	EC, EN	o.k.		
SOMCP	50	0.0049		o.k. / Interaction with	Loose bowel motions in three weeks before 1st interview	0.0189
AE	13600	0.0370	CP, EC, EN	o.k.		
PA	---					
**Gastroenteritis (Definition NL-2)**						
EC	2163	0.0030	CP, EN	o.k.		
IE	145	0.0018		o.k. / Interaction with	Stomach pains or cramps in three weeks before 1st interview	0.0084
IE				o.k. / Interaction with	Proneness to motion sickness	0.0152
CP	36	0.0032		o.k. / Interaction with	Stomach pains or cramps in three weeks before 1st interview	0.0234
SOMCP	119	0.0180		o.k. / Interaction with	Stomach pains or cramps in three weeks before 1st interview	0.0319
AE	---					
PA	---					
**Skin inflammation, cutireaction**						
EC	n. a.					
IE	n. a.					
CP	n. a.					
SOMCP	n. a.					
AE	n. a.					
PA	n. a.					

Supplemental Data, Annex 7. (continued).

**Disease** Indicator	Potential NOAEL	*p*-Value [a]	Better predictor [b]	Status	Confounding or interacting variable	*p*-Value [c]
**Urinary tract infection**						
EC	n. a.					
IE	n. a.					
CP	n. a.					
SOMCP	n. a.					
AE	n. a.					
PA	n. a.					

Abbreviations: EC, Escherichia coli; IE, intestinal enterococci; CP, Clostridium perfringens; SOMCP, somatic coliphages; AE, aeromonads; PA, Pseudomonas aeruginosa; NOAEL, No Observed Adverse Effect Level (Microorganisms/100 mL); o.k., not confounded by any of the tested potential confounding variables; [a] Chi Square *p*-Value in a Pearson's Chi Square test comparing the incidence rates of disease below and above the potential NOAEL. [b] Better predictors are indicator organisms which are significantly better predictors ($p<0.05$) of the outcome (disease) in an Effect Likelihood Ratio Test modeling the disease as response variable and the NOAELs of two indicator organisms as model effects. [c] Chi Square *p*-value for the potential confounding or interacting variable in an Effect Likelihood Ratio Test modeling the disease as response variable and the potential NOAEL and the potential confounding variable as model effects. [d] rounded. --- No concentration fulfilling the validity criteria for a potential NOAEL.

Supplemental Data, Annex 8. Incidence rates of gastroenteritis and influence of swallowing water below and above NOAELs of fecal indicator organisms.

Exposure Definition 1: "10 minutes bathing with at least 3 head immersions"[i]

***Escherichia coli*/100mL by gastroenteritis definition "UK"[j]**

NOAEL [a] (MO/100mL)	Exposure Status	Cases	n	IR [b] (%)	*p*-Value [c]	RR [d]	95% CI [LL; UL]	aR [e]	"SW?"	Cases	n	IR (%)	*p*-Value	aR [f]
	Unexposed	13	920	1.4						13	920	1.4		
180	Exposed ≤ NOAEL	7	409	1.7	0.68				"no"	3	247	1.2	1.00	
									"yes"	4	162	2.5	0.30	1.3
	Exposed > NOAEL	21	419	5.0	0.009 **	3.5	[1.8; 7.0]	3.6	"no"	6	198	3.0	0.13	
									"yes"	15	217	6.9	<0.001***	3.9
	Total	41	1748							41	1744			

Exposure Definition 1: "10 minutes bathing with at least 3 head immersions"

***Escherichia coli*/100mL by gastroenteritis definition "UK-wf"**

NOAEL [a] (MO/100mL)	Exposure Status	Cases	n	IR [b] (%)	*p*-Value [c]	RR [d]	95% CI [LL; UL]	aR [e]	"SW?"	Cases	n	IR (%)	*p*-Value	aR [f]
	Unexposed	26	921	2.8						26	921	2.8		
78	Exposed ≤ NOAEL	5	259	1.9	0.43				"no"	2	155	1.3	0.41	
									"yes"	3	104	2.9	1.00	1.6
	Exposed > NOAEL	41	579	7.1	0.002 **	2.5	[1.6; 4.0]	4.3	"no"	17	296	5.7	0.02 *	
									"yes"	24	279	8.6	<0.001***	2.9
	Total	72	1759							72	1755			

Exposure Definition 1: "10 minutes bathing with at least 3 head immersions"

***Escherichia coli*/100mL by gastroenteritis definition "NL-2"**

NOAEL [a] (MO/100mL)	Exposure Status	Cases	n	IR [b] (%)	*p*-Value [c]	RR [d]	95% CI [LL; UL]	aR [e]	"SW?"	Cases	n	IR (%)	*p*-Value	aR [f]
	Unexposed	48	921	5.2						48	921	5.2		
167	Exposed ≤ NOAEL	18	396	4.5	0.61				"no"	10	241	4.1	0.50	
									"yes"	8	155	5.2	0.98	1.0
	Exposed > NOAEL	45	441	10.2	0.002 **	2.0	[1.3; 2.9]	5.0	"no"	18	210	8.6	0.06	
									"yes"	27	227	11.9	<0.001***	3.3
	Total	111	1758							111	1754			

NOAEL [a] (MO/100mL)	Exposure Status	Cases	n	IR [b] (%)	*p*-Value [c]	RR [d]	95% CI [LL; UL]	aR [e]	"SW?"	Cases	n	IR (%)	*p*-Value	aR [f]
**Exposure Definition 1: "10 minutes bathing with at least 3 head immersions"**														
**Intestinal enterococci/100mL by gastroenteritis definition "UK"**														
	Unexposed	13	920	1.4						13	920	1.4		
24	Exposed ≤ NOAEL	5	320	1.6	0.79				"no"	2	189	1.1	1.00	
									"yes"	3	129	2.3	0.43	1.3
	Exposed > NOAEL	23	508	4.5	0.02 *	3.2	[1.6; 6.3]	3.1	"no"	7	256	2.7	0.17	
									"yes"	16	250	6.4	<0.001***	3.7
	Total	41	1748							41	1744			
**Exposure Definition 1: "10 minutes bathing with at least 3 head immersions"**														
**Intestinal enterococci/100mL by gastroenteritis definition "UK-wf"**														
	Unexposed	26	921	2.8						26	921	2.8		
21	Exposed ≤ NOAEL	6	307	2.0	0.41				"no"	3	181	1.7	0.61	
									"yes"	3	126	2.4	1.00	0.7
	Exposed > NOAEL	40	531	7.5	<0.001***	2.7	[1.7; 4.3]	4.7	"no"	16	270	5.9	0.02 *	
									"yes"	24	257	9.3	<0.001***	3.4
	Total	72	1759							72	1755			
**Exposure Definition 1: "10 minutes bathing with at least 3 head immersions"**														
**Intestinal enterococci/100mL by gastroenteritis definition "NL-2"**														
	Unexposed	48	921	5.2						48	921	5.2		
24	Exposed ≤ NOAEL	12	321	3.7	0.29				"no"	5	190	2.6	0.13	
									"yes"	7	129	5.4	0.92	2.8
	Exposed > NOAEL	51	516	9.9	0.001 **	1.9	[1.3; 2.8]	4.7	"no"	23	261	8.8	0.03 *	
									"yes"	28	253	11.1	<0.001***	2.3
	Total	111	1758							111	1754			

NOAEL [a] (MO/100mL)	Exposure Status	Cases	n	IR [b] (%)	*p*-Value [c]	RR [d]	95% CI [LL; UL]	aR [e]	"SW?"	Cases	n	IR (%)	*p*-Value	aR [f]
**Exposure Definition 1: "10 minutes bathing with at least 3 head immersions"**														
***Clostridium perfringens*/100mL by gastroenteritis definition "UK"**														
	Unexposed	13	920	1.4						13	920	1.4		
13	Exposed ≤ NOAEL	4	329	1.2	1.00				"no"	2	192	1.0	1.00	
									"yes"	2	135	1.5	1.00	0.4
	Exposed > NOAEL	24	508	4.7	0.006 **	3.3	[1.7; 6.5]	3.3	"no"	7	256	2.7	0.17	
									"yes"	17	250	6.8	<0.001***	4.1
	Total	41	1757							41	1753			
**Exposure Definition 1: "10 minutes bathing with at least 3 head immersions"**														
***Clostridium perfringens*/100mL by gastroenteritis definition "UK-wf"**														
	Unexposed	26	921	2.8						26	921	2.8		
13	Exposed ≤ NOAEL	8	331	2.4	0.70				"no"	4	193	2.1	0.56	
									"yes"	4	136	2.9	1.00	0.9
	Exposed > NOAEL	38	516	7.4	0.002 **	2.6	[1.6; 4.3]	4.5	"no"	15	261	5.7	0.02 *	
									"yes"	23	253	9.1	<0.001***	3.3
	Total	72	1768							72	1764			
**Exposure Definition 1: "10 minutes bathing with at least 3 head immersions"**														
***Clostridium perfringens*/100mL by gastroenteritis definition "NL-2"**														
	Unexposed	48	921	5.2						48	921	5.2		
13	Exposed ≤ NOAEL	13	331	3.9	0.35				"no"	7	193	3.6	0.36	
									"yes"	6	136	4.4	0.69	0.8
	Exposed > NOAEL	50	515	9.7	0.002 **	1.9	[1.3; 2.7]	4.5	"no"	21	261	8.0	0.08	
									"yes"	29	252	11.5	<0.001***	3.5
	Total	111	1767							111	1763			

NOAEL [a] (MO/100mL)	Exposure Status	Cases	n	IR [b] (%)	*p*-Value [c]	RR [d]	95% CI [LL; UL]	aR [e]	"SW?"	Cases	n	IR (%)	*p*-Value	aR [f]
**Exposure Definition 1: "10 minutes bathing with at least 3 head immersions"**														
**Somatic coliphages/100mL by gastroenteritis definition "UK"**														
	Unexposed	13	920	1.4						13	920	1.4		
150	Exposed ≤ NOAEL	16	656	2.4	0.14				"no"	7	369	1.9	0.53	
									"yes"	9	285	3.2	0.05	1.3
	Exposed > NOAEL	11	169	6.5	0.008 **	4.6	[2.1; 10.1]	5.1	"no"	2	72	2.8	0.30	
									"yes"	9	95	9.5	<0.001***	6.7
	Total	40	1745							40	1741			
**Exposure Definition 1: "10 minutes bathing with at least 3 head immersions"**														
**Somatic coliphages/100mL by gastroenteritis definition "UK-wf"**														
	Unexposed	26	921	2.8						26	921	2.8		
10	Exposed ≤ NOAEL	7	305	2.3	0.62				"no"	3	180	1.7	0.61	
									"yes"	4	123	3.3	0.77	1.6
	Exposed > NOAEL	37	530	7.0	0.004 **	2.5	[1.5; 4.0]	4.2	"no"	15	267	5.6	0.03 *	
									"yes"	22	261	8.4	<0.001***	2.8
	Total	70	1756							70	1752			
**Exposure Definition 1: "10 minutes bathing with at least 3 head immersions"**														
**Somatic coliphages/100mL by gastroenteritis definition "NL-2"**														
	Unexposed	48	921	5.2						48	921	5.2		
10	Exposed ≤ NOAEL	11	304	3.6	0.26				"no"	5	180	2.8	0.16	
									"yes"	6	122	4.9	0.89	2.1
	Exposed > NOAEL	49	530	9.2	0.002 **	1.8	[1.2; 2.6]	4.0	"no"	22	267	8.2	0.06	
									"yes"	27	261	10.3	0.003 **	2.1
	Total	108	1755							108	1751			

Exposure Definition 2: "Single head immersion"
***Escherichia coli*/100mL by gastroenteritis definition "UK"**

NOAEL [a] (MO/100mL)	Exposure Status	Cases	n	IR [b] (%)	*p*-Value [c]	RR [d]	95% CI [LL; UL]	aR [e]	"SW?"	Cases	n	IR (%)	*p*-Value	aR [f]
	Unexposed	13	920	1.4						13	920	1.4		
1453	Exposed ≤ NOAEL	11	555	2.0	0.40				"no"	4	308	1.3	1.00	
									"yes"	7	245	2.9	0.16	1.6
	Exposed > NOAEL	17	273	6.2	0.001 **	4.4	[2.2; 8.7]	4.8	"no"	5	137	3.6	0.07	
									"yes"	12	134	9.0	<0.001***	5.3
	Total	41	1748							41	1744			

Exposure Definition 2: "Single head immersion"
***Escherichia coli*/100mL by gastroenteritis definition "UK-wf"**

Not applicable. No valid NOAEL detected.

Exposure Definition 2: "Single head immersion"
***Escherichia coli*/100mL by gastroenteritis definition "NL-2"**

NOAEL [a] (MO/100mL)	Exposure Status	Cases	n	IR [b] (%)	*p*-Value [c]	RR [d]	95% CI [LL; UL]	aR [e]	"SW?"	Cases	n	IR (%)	*p*-Value	aR [f]
	Unexposed	48	921	5.2						48	921	5.2		
2163	Exposed ≤ NOAEL	37	623	5.9	0.54				"no"	20	348	5.7	0.71	
									"yes"	17	272	6.3	0.51	0.5
	Exposed > NOAEL	26	214	12.1	0.003 **	2.3	[1.5; 3.7]	6.9	"no"	8	103	7.8	0.28	
									"yes"	18	110	16.4	<0.001***	8.6
	Total	111	1758							111	1754			

NOAEL [a] (MO/100mL)	Exposure Status	Cases	n	IR [b] (%)	*p*-Value [c]	RR [d]	95% CI [LL; UL]	aR [e]	"SW?"	Cases	n	IR (%)	*p*-Value	aR [f]
**Exposure Definition 2: "Single head immersion"**														
**Intestinal enterococci/100mL by gastroenteritis definition "UK"**														
	Unexposed	13	920	1.4						13	920	1.4		
123	Exposed ≤ NOAEL	5	351	1.4	1.00				"no"	2	203	1.0	1.00	
									"yes"	3	146	2.1	0.47	1.1
	Exposed > NOAEL	23	477	4.8	0.008 **	3.4	[1.7; 6.7]	3.4	"no"	7	242	2.9	0.16	
									"yes"	16	233	6.9	<0.001***	4.0
	Total	41	1748							41	1744			
**Exposure Definition 2: "Single head immersion"**														
**Intestinal enterococci/100mL by gastroenteritis definition "UK-wf"**														
	Unexposed	26	921	2.8						26	921	2.8		
123	Exposed ≤ NOAEL	8	353	2.3	0.58				"no"	3	204	1.5	0.27	
									"yes"	5	147	3.4	0.60	1.9
	Exposed > NOAEL	38	485	7.8	<0.001***	2.8	[1.7; 4.5]	5.0	"no"	16	247	6.5	0.006 **	
									"yes"	22	236	9.3	<0.001***	2.8
	Total	72	1759							72	1755			
**Exposure Definition 2: "Single head immersion"**														
**Intestinal enterococci/100mL by gastroenteritis definition "NL-2"**														
	Unexposed	48	921	5.2						48	921	5.2		
124	Exposed ≤ NOAEL	16	370	4.3	0.51				"no"	6	216	2.8	0.13	
									"yes"	10	153	6.5	0.50	3.8
	Exposed > NOAEL	47	467	10.1	0.002 **	1.9	[1.3; 2.8]	4.9	"no"	22	235	9.4	0.02 *	
									"yes"	25	229	10.9	0.002 **	1.6
	Total	111	1758							111	1754			

NOAEL [a] (MO/100mL)	Exposure Status	Cases	n	IR [b] (%)	*p*-Value [c]	RR [d]	95% CI [LL; UL]	aR [e]	"SW?"	Cases	n	IR (%)	*p*-Value	aR [f]
**Exposure Definition 2: "Single head immersion"**														
***Clostridium perfringens*/100mL by gastroenteritis definition "UK"**														
	Unexposed	13	920	1.4						13	920	1.4		
38	Exposed ≤ NOAEL	3	249	1.2	1.00				"no"	2	145	1.4	1.00	
									"yes"	1	104	1.0	1.00	-0.4
	Exposed > NOAEL	25	588	4.3	0.03 *	3.0	[1.6; 5.8]	2.8	"no"	7	303	2.3	0.30	
									"yes"	18	281	6.4	<0.001***	4.1
	Total	41	1757							41	1753			
**Exposure Definition 2: "Single head immersion"**														
***Clostridium perfringens*/100mL by gastroenteritis definition "UK-wf"**														
	Unexposed	26	921	2.8						26	921	2.8		
38	Exposed ≤ NOAEL	6	251	2.4	0.71				"no"	3	146	2.1	0.79	
									"yes"	3	105	2.9	1.00	0.8
	Exposed > NOAEL	40	596	6.7	0.01 *	2.4	[1.5; 3.9]	3.9	"no"	16	308	5.2	0.05 *	
									"yes"	24	284	8.5	<0.001***	3.3
	Total	72	1768							72	1764			
**Exposure Definition 2: "Single head immersion"**														
***Clostridium perfringens*/100mL by gastroenteritis definition "NL-2"**														
	Unexposed	48	921	5.2						48	921	5.2		
36	Exposed ≤ NOAEL	5	194	2.6	0.12				"no"	3	117	2.6	0.21	
									"yes"	2	77	2.6	0.42	0.0
	Exposed > NOAEL	58	652	8.9	0.003 **	1.7	[1.2; 2.5]	3.7	"no"	25	337	7.4	0.14	
									"yes"	33	311	10.6	<0.001***	3.2
	Total	111	1767							111	1763			

NOAEL [a] (MO/100mL)	Exposure Status	Cases	n	IR [b] (%)	*p*-Value [c]	RR [d]	95% CI [LL; UL]	aR [e]	"SW?"	Cases	n	IR (%)	*p*-Value	aR [f]
**Exposure Definition 2: "Single head immersion"**														
**Somatic coliphages/100mL by gastroenteritis definition "UK"**														
	Unexposed	13	920	1.4						13	920	1.4		
330	Exposed ≤ NOAEL	9	487	1.8	0.53				"no"	4	299	1.3	1.00	
									"yes"	5	186	2.7	0.21	1.4
	Exposed > NOAEL	18	338	5.3	0.006 **	3.8	[1.9; 7.6]	3.9	"no"	5	142	3.5	0.08	
									"yes"	13	194	6.7	<0.001***	3.2
	Total	40	1745							40	1741			
**Exposure Definition 2: "Single head immersion"**														
**Somatic coliphages/100mL by gastroenteritis definition "UK-wf"**														
	Unexposed	26	921	2.8						26	921	2.8		
50	Exposed ≤ NOAEL	7	298	2.3	0.66				"no"	3	179	1.7	0.61	
									"yes"	4	118	3.4	0.77	1.7
	Exposed > NOAEL	37	537	6.9	0.005 **	2.4	[1.5; 4.0]	4.1	"no"	15	268	5.6	0.03 *	
									"yes"	22	266	8.3	<0.001***	2.7
	Total	70	1756							70	1752			
**Exposure Definition 2: "Single head immersion"**														
**Somatic coliphages/100mL by gastroenteritis definition "NL-2"**														
	Unexposed	48	921	5.2						48	921	5.2		
119	Exposed ≤ NOAEL	19	386	4.9	0.83				"no"	11	236	4.7	0.73	
									"yes"	8	149	5.4	0.94	0.7
	Exposed > NOAEL	41	448	9.2	0.02 *	1.8	[1.2; 2.6]	3.9	"no"	16	211	7.6	0.18	
									"yes"	25	234	10.7	0.002 **	3.1
	Total	108	1755							108	1751			

Abbreviations: NOAEL, No Observed Adverse Effect Level; MO/100mL, microorganisms per 100 mL; n, total number of participants per category; IR, incidence rate; RR, relative risk; CI, confidence interval; LL, lower limit; UL, upper limit; aR, attributable risk; "SW?", "Did you swallow water?"; UK, definition of gastroenteritis used in the United Kingdom according to Kay et al., 1994 ((diarrhea with three or more bowl movements per day) or vomiting or (nausea and fever) or (indigestion and fever)); UK-wf; UK definition without consideration of stool frequency (diarrhea or vomiting or (nausea and fever) or (indigestion and fever)); NL-2; definition of gastroenteritis used in the Netherlands according to van Asperen et al., 1998 (diarrhea or vomiting or nausea or stomach pains). [a] NOAELs were determined as the concentration yielding the minimal *p*-value in a series of Pearson's Chi Square tests comparing the incidence rates of disease of bathers exposed at or below a certain exposure concentration with the incidence rate of bathers exposed above this concentration for all actually occurring exposure concentrations. [b] Incidence rate (%) is the percentage of individuals with onset of disease within one week after exposure per category. [c] The *p*-value was calculated by a Pearson's chi-square test or Fisher's exact test that compared the number of cases among the bathers who were exposed in one of the given categories of fecal indicator concentrations with the number of cases among the unexposed participants (non-bathers). Fisher's exact test results were used when an expected cell value was less than five. [d] Relative risk is the incidence rate of disease among bathers exposed above NOAEL divided by the incidence rate of disease among non-bathers one week after exposure. [e] Attributable risk of bathing above NOAEL is the incidence rate of disease (in %) among bathers exposed above NOAEL minus the incidence rate of non-bathers. [f] Attributable risk of swallowing water below NOAEL is the incidence rate of disease among bathers exposed at or below NOAEL and reporting to have swallowed water minus the incidence rate of disease among bathers exposed at or below NOAEL and reporting not to have swallowed water. Attributable risk of swallowing water above NOAEL is the incidence rate of disease among bathers exposed above NOAEL and reporting to have swallowed water minus the incidence rate of disease among bathers exposed above NOAEL and reporting not to have swallowed water.* *p*-Value <0.05. ** *p*-Value <0.01. *** *p*-Value <0.001.

Supplemental Data, Annex 9: Incidence rates of gastroenteritis (definition UK-wf) in quartile and quintile categories of microbial exposure concentrations for exposure definition 1 ("10 minutes bathing with at least three head immersions").

Parameter	Quartile UL (MO/100mL)	Cases	n	IR [a] (%)	*p*-Value [b]		Quintile UL	Cases	n	IR (%)	*p*-Value	
**GE definition "UK"**												
	Unexposed	13	920	1.4			unexposed	13	920	1.4		
Escherichia coli	72	2	205	1.0	1.00		61	1	164	0.6	0.71	
	181	6	209	2.9	0.14		116	5	167	3.0	0.18	
	379	8	207	3.9	0.04	*	245	5	167	3.0	0.18	
	4600	12	207	5.8	<0.001	***	445	7	163	4.3	0.02	*
							4600	10	167	6.0	0.001	***
**GE definition "UK-wf"**												
	Unexposed	26	921	2.8			unexposed	26	921	2.8		
Escherichia coli	72	4	207	1.9	0.47		61	3	166	1.8	0.61	
	181	11	212	5.2	0.08		116	6	168	3.6	0.62	
	379	14	211	6.6	0.007	**	245	10	170	5.9	0.04	*
	4600	17	208	8.2	<0.001	***	445	12	166	7.2	0.004	**
							4600	15	168	8.9	<0.001	***
**GE definition "NL-2"**												
	Unexposed	48	921	5.2			unexposed	48	921	5.2		
Escherichia coli	72	7	207	3.4	0.27		61	6	166	3.6	0.38	
	181	14	211	6.6	0.41		116	9	167	5.4	0.92	
	379	22	211	10.4	0.005	**	245	15	170	8.8	0.06	
	4600	20	208	9.6	0.02	*	445	16	166	9.6	0.03	*
							4600	17	168	10.1	0.01	*

Supplemental Data, Annex 9: (continued).

Parameter	Quartile UL (MO/100mL)	Cases	n	IR [a] (%)	*p*-Value [b]		Quintile UL	Cases	n	IR (%)	*p*-Value	
**GE definition "UK"**												
	Unexposed	13	920	1.4			unexposed	13	920	1.4		
Intestinal enterococci	14	4	207	1.9	0.53		12	3	167	1.8	0.72	
	53	5	210	2.4	0.35		27	3	165	1.8	0.72	
	101	10	207	4.8	0.004	**	68	11	167	6.6	<0.001	***
	1190	9	204	4.4	0.01	*	114	4	166	2.4	0.31	
							1190	7	163	4.3	0.02	*
**GE definition "UK-wf"**												
	Unexposed	26	921	2.8			unexposed	26	921	2.8		
Intestinal enterococci	14	5	208	2.4	0.74		12	3	167	1.8	0.61	
	53	9	212	4.2	0.28		27	5	167	3.0	0.80	
	101	14	210	6.7	0.007	**	68	15	169	8.9	<0.001	***
	1190	18	208	8.7	<0.001	***	114	7	168	4.2	0.35	
							1190	16	167	9.6	<0.001	***
**GE definition "NL-2"**												
	Unexposed	48	921	5.2			unexposed	48	921	5.2		
Intestinal enterococci	14	8	208	3.8	0.41		12	5	167	3.0	0.22	
	53	13	211	6.2	0.58		27	10	166	6.0	0.67	
	101	19	210	9.0	0.03	*	68	18	169	10.7	0.006	**
	1190	23	208	11.1	0.002	**	114	9	168	5.4	0.94	
							1190	21	167	12.6	<0.001	***

Supplemental Data, Annex 9: (continued).

Parameter	Quartile UL (MO/100mL)	Cases	n	IR [a] (%)	*p*-Value [b]		Quintile UL	Cases	n	IR (%)	*p*-Value	
**GE definition "UK"**												
	Unexposed	13	920	1.4			unexposed	13	920	1.4		
Clostridium perfringens	9	4	222	1.8	0.76		9	4	222	1.8	0.76	
	18	4	199	2.0	0.52		13	2	115	1.7	0.68	
	33	11	209	5.3	0.002	**	22	7	169	4.1	0.02	*
	148	9	207	4.3	0.01	*	36	9	164	5.5	0.003	**
							148	6	167	3.6	0.06	
**GE definition "UK-wf"**												
	Unexposed	26	921	2.8			unexposed	26	921	2.8		
Clostridium perfringens	9	6	224	2.7	0.91		9	6	224	2.7	0.91	
	18	9	202	4.5	0.23		13	4	115	3.5	0.57	
	33	18	211	8.5	<0.001	***	22	12	172	7.0	0.006	**
	148	13	210	6.2	0.02	*	36	14	166	8.4	<0.001	***
							148	10	170	5.9	0.04	*
**GE definition "NL-2"**												
	Unexposed	48	921	5.2			unexposed	48	921	5.2		
Clostridium perfringens	9	11	224	4.9	0.85		9	11	224	4.9	0.86	
	18	12	202	5.9	0.68		13	4	115	3.5	0.42	
	33	23	211	10.9	0.002	*	22	17	172	9.9	0.02	*
	148	17	209	8.1	0.10		36	19	165	11.5	0.002	*
							148	12	170	7.1	0.33	

Supplemental Data, Annex 9: (continued).

Parameter	Quartile UL (MO/100mL)	Cases	n	IR [a] (%)	*p*-Value [b]		Quintile UL	Cases	n	IR (%)	*p*-Value	
**GE definition "UK"**												
	Unexposed	13	920	1.4			unexposed	13	920	1.4		
Somatic coliphages	10	5	300	1.7	0.78		10	5	300	1.7	0.78	
	35	4	111	3.6	0.10		11	2	30	6.7	0.08	
	142	7	209	3.3	0.08		85	5	163	3.1	0.17	
	3598	11	205	5.4	0.002	**	153	5	169	3.0	0.18	
							3598	10	163	6.1	<0.001	***
**GE definition "UK-wf"**												
	Unexposed	26	921	2.8			unexposed	26	921	2.8		
Somatic coliphages	10	7	302	2.3	0.64		10	7	302	2.3	0.64	
	35	9	115	7.8	0.01	*	11	3	31	9.7	0.06	
	142	12	212	5.7	0.04	*	85	11	167	6.6	0.01	*
	3598	16	206	7.8	<0.001	***	153	10	171	5.8	0.04	*
							3598	13	164	7.9	0.001	***
**GE definition "NL-2"**												
	Unexposed	48	921	5.2			unexposed	48	921	5.2		
Somatic coliphages	10	11	301	3.7	0.27		10	11	301	3.7	0.27	
	35	12	115	10.4	0.02	*	11	4	31	12.9	0.08	
	142	17	212	8.0	0.11		85	14	167	8.4	0.10	
	3598	20	206	9.7	0.01	*	153	15	171	8.8	0.07	
							3598	16	164	9.8	0.02	*

Abbreviations: GE, gastroenteritis; UK-wf, definition of gastroenteritis used in the United Kingdom according to Kay et al., 1994, but without consideration of stool frequency (diarrhea or vomiting or nausea and fever or indigestion and fever); UL, Upper limit of quantile range; MO/100mL, microorganisms per 100 mL; n, total number of participants per category; IR, incidence rate. [a] Incidence rate (%) is the percentage of individuals with onset of disease within one week after exposure per category. [b] The *p*-value was calculated by a Pearson's chi-square test or Fisher's exact test that compared the number of cases among the bathers who were exposed in one of the given categories of fecal indicator concentrations with the number of cases among the unexposed participants (non-bathers). Fisher's exact test results were used when an expected cell value was less than five. * *p*-Value <0.05. ** *p*-Value <0.01. *** *p*-Value <0.001.

Supplemental Data, Annex 10: Incidence rates of gastroenteritis (definition UK-wf) in quartile and quintile categories of microbial exposure concentrations for exposure definition 2 ("Single head immersion").

Parameter	Quartile UL (MO/100mL)	Cases	n	IR [a] (%)	*p*-Value [b]		Quintile UL	Cases	n	IR (%)	*p*-Value	
**GE definition "UK"**												
	Unexposed	13	920	1.4			unexposed	13	920	1.4		
Escherichia coli	233	3	205	1.5	1.00		206	0	164	0.0	0.24	
	739	4	209	1.9	0.54		471	7	166	4.2	0.02	*
	2204	10	208	4.8	0.005	**	1086	2	165	1.2	1.00	
	32812	11	206	5.3	0.002	**	2749	12	167	7.2	<0.001	***
							32812	7	166	4.2	0.02	*
**GE definition "UK-wf"**												
	Unexposed	26	921	2.8			unexposed	26	921	2.8		
Escherichia coli	233	4	206	1.9	0.48		206	1	165	0.6	0.11	
	739	11	213	5.2	0.08		471	10	169	5.9	0.04	*
	2204	13	210	6.2	0.02	*	1086	8	168	4.8	0.18	
	32812	18	209	8.6	<0.001	***	2749	16	169	9.5	<0.001	***
							32812	11	167	6.6	0.01	*
**GE definition "NL-2"**												
	Unexposed	48	921	5.2			unexposed	48	921	5.2		
Escherichia coli	233	8	206	3.9	0.43		206	4	165	2.4	0.12	
	739	16	212	7.5	0.18		471	13	168	7.7	0.19	
	2204	15	210	7.1	0.27		1086	13	168	7.7	0.19	
	32812	24	209	11.5	<0.001	***	2749	19	169	11.2	0.003	**
							32812	14	167	8.4	0.10	

Supplemental Data, Annex 10: (continued).

Parameter	Quartile UL (MO/100mL)	Cases	n	IR [a] (%)	*p*-Value [b]		Quintile UL	Cases	n	IR (%)	*p*-Value	
**GE definition "UK"**												
	Unexposed	13	920	1.4			unexposed	13	920	1.4		
Intestinal enterococci	53	4	208	1.9	0.53		42	2	168	1.2	1.00	
	192	8	207	3.9	0.04	*	112	3	164	1.8	0.72	
	427	7	209	3.3	0.08		256	9	167	5.4	0.003	**
	11816	9	204	4.4	0.01	*	545	6	166	3.6	0.06	*
							11816	8	163	4.9	0.008	**
**GE definition "UK-wf"**												
	Unexposed	26	921	2.8			unexposed	26	921	2.8		
Intestinal enterococci	53	4	208	1.9	0.47		42	2	168	1.2	0.29	
	192	12	210	5.7	0.04	*	112	6	166	3.6	0.62	
	427	13	212	6.1	0.02	*	256	11	168	6.5	0.01	*
	11816	17	208	8.2	<0.001	***	545	12	170	7.1	0.006	**
							11816	15	166	9.0	<0.001	***
**GE definition "NL-2"**												
	Unexposed	48	921	5.2			unexposed	48	921	5.2		
Intestinal enterococci	53	7	208	3.4	0.26		42	4	168	2.4	0.11	
	192	17	209	8.1	0.10		112	11	165	6.7	0.45	
	427	16	212	7.5	0.18		256	12	168	7.1	0.31	
	11816	23	208	11.1	0.002	**	545	17	170	10.0	0.02	*
							11816	19	166	11.4	0.002	**

Supplemental Data, Annex 10: (continued).

Parameter	Quartile UL (MO/100mL)	Cases	n	IR [a] (%)	*p*-Value [b]		Quintile UL	Cases	n	IR (%)	*p*-Value	
**GE definition "UK"**												
	Unexposed	13	920	1.4			unexposed	13	920	1.4		
Clostridium perfringens	36	2	242	0.8	1.00		27	1	168	0.6	0.71	
	73	8	185	4.3	0.02	*	55	5	174	2.9	0.19	
	146	9	201	4.5	0.009	**	99	5	161	3.1	0.17	
	3600	9	209	4.3	0.001	**	175	10	168	6.0	0.001	**
							3600	7	166	4.2	0.02	*
**GE definition "UK-wf"**												
	Unexposed	26	921	2.8			unexposed	26	921	2.8		
Clostridium perfringens	36	5	244	2.0	0.50		27	3	169	1.8	0.61	
	73	11	186	5.9	0.03	*	55	9	176	5.1	0.11	
	146	17	205	8.3	<0.001	***	99	7	162	4.3	0.32	
	3600	13	212	6.1	0.02	*	175	16	171	9.4	<0.001	***
							3600	11	169	6.5	0.02	*
**GE definition "NL-2"**												
	Unexposed	48	921	5.2			unexposed	48	921	5.2		
Clostridium perfringens	36	10	244	4.1	0.48		27	5	169	3.0	0.21	
	73	15	186	8.1	0.13		55	15	176	8.5	0.08	
	146	22	205	10.7	0.003	**	99	8	162	4.9	0.88	
	3600	16	211	7.6	0.18		175	22	170	12.9	<0.001	***
							3600	13	169	7.7	0.20	

Supplemental Data, Annex 10: (continued).

Parameter	Quartile UL (MO/100mL)	Cases	n	IR [a] (%)	*p*-Value [b]		Quintile UL	Cases	n	IR (%)	*p*-Value	
**GE definition "UK"**												
	Unexposed	13	920	1.4			unexposed	13	920	1.4		
Somatic coliphages	40	5	252	2.0	0.56		30	4	177	2.3	0.50	
	184	4	159	2.5	0.93		66	2	151	1.3	1.00	
	674	8	206	3.9	0.04	*	350	5	165	3.0	0.17	
	28615	10	208	4.8	0.005	**	959	7	166	4.2	0.02	*
							28615	9	166	5.4	0.003	**
**GE definition "UK-wf"**												
	Unexposed	26	921	2.8			unexposed	26	921	2.8		
Somatic coliphages	40	7	254	2.8	0.95		30	5	178	2.8	0.99	
	184	9	162	5.6	0.07		66	6	155	3.9	0.45	
	674	15	210	7.1	0.003	*	350	10	167	6.0	0.04	*
	28615	13	209	6.2	0.02	*	959	11	168	6.5	0.01	*
							28615	12	167	7.2	0.005	*
**GE definition "NL-2"**												
	Unexposed	48	921	5.2			unexposed	48	921	5.2		
Somatic coliphages	40	12	254	4.7	0.75		30	8	178	4.5	0.69	
	184	11	161	6.8	0.40		66	9	154	5.8	0.75	
	674	20	210	9.5	0.02	*	350	13	167	7.8	0.18	
	28615	17	209	8.1	0.10		959	15	168	8.9	0.06	
							28615	15	167	9.0	0.05	

Abbreviations: GE, gastroenteritis; UK-wf, definition of gastroenteritis used in the United Kingdom according to Kay et al., 1994, but without consideration of stool frequency (diarrhea or vomiting or nausea and fever or indigestion and fever); UL, Upper limit of quantile range; MO/100mL, microorganisms per 100 mL; n, total number of participants per category; IR, incidence rate. [a] Incidence rate (%) is the percentage of individuals with onset of disease within one week after exposure per category. [b] The *p*-value was calculated by a Pearson's chi-square test or Fisher's exact test that compared the number of cases among the bathers who were exposed in one of the given categories of fecal indicator concentrations with the number of cases among the unexposed participants (non-bathers). Fisher's exact test results were used when an expected cell value was less than five. * *p*-Value <0.05. ** *p*-Value <0.01. *** *p*-Value <0.001.
